# FOXN1 forms higher-order nuclear condensates displaced by mutations causing immunodeficiency

**DOI:** 10.1126/sciadv.abj9247

**Published:** 2021-12-03

**Authors:** Ioanna A. Rota, Adam E. Handel, Stefano Maio, Fabian Klein, Fatima Dhalla, Mary E. Deadman, Stanley Cheuk, Joseph A. Newman, Yale S. Michaels, Saulius Zuklys, Nicolas Prevot, Philip Hublitz, Philip D. Charles, Athina Soragia Gkazi, Eleni Adamopoulou, Waseem Qasim, Edward Graham Davies, Imelda Hanson, Alistair T. Pagnamenta, Carme Camps, Helene M. Dreau, Andrea White, Kieran James, Roman Fischer, Opher Gileadi, Jenny C. Taylor, Tudor Fulga, B. Christoffer Lagerholm, Graham Anderson, Erdinc Sezgin, Georg A. Holländer

**Affiliations:** 1 Department of Paediatrics and the MRC Weatherall Institute of Molecular Medicine, University of Oxford, Oxford, UK.; 2 Nuffield Department of Clinical Neurosciences, University of Oxford, Oxford, UK.; 3 Structural Genomics Consortium, University of Oxford, ORCRB, Roosevelt Drive, Oxford, UK.; 4 Genome Engineering and Synthetic Biology Unit, MRC Weatherall Institute of Molecular Medicine, University of Oxford, Oxford, UK.; 5 Paediatric Immunology, Department of Biomedicine, University of Basel and University Children’s Hospital Basel, Basel, Switzerland.; 6 MRC Weatherall Institute of Molecular Medicine, Genome engineering services, Radcliffe Department of Medicine, University of Oxford, Oxford, UK.; 7 Target Discovery Institute, University of Oxford, Oxford OX3 7FZ, UK.; 8 Great Ormond Street Hospital and Great Ormond Street Institute of Child Health, University College London, London WC1N 1EH, UK.; 9 Department of Pediatrics, Section of Pediatric Immunology, Allergy, and Retrovirology, Baylor College of Medicine, Houston, TX, USA.; 10 National Institute for Health Research Biomedical Research Centre, Oxford, UK.; 11 Wellcome Centre for Human Genetics, University of Oxford, Oxford OX3 7BN, UK.; 12 Department of Oncology, University of Oxford, Oxford OX3 7DQ, UK.; 13 Institute for Immunology and Immunotherapy, Medical School, University of Birmingham, Birmingham B15 2TT, UK.; 14 Wolfson Imaging Centre Oxford, MRC Weatherall Institute of Molecular Medicine, University of Oxford, Headley Way, Oxford OX3 9DS, UK.; 15 MRC Human Immunology Unit, MRC Weatherall Institute of Molecular Medicine, University of Oxford, Oxford, UK.; 16 Department of Biosystems Science and Engineering, ETH Zurich, Basel, Switzerland.

## Abstract

The transcription factor FOXN1 is a master regulator of thymic epithelial cell (TEC) development and function. Here, we demonstrate that FOXN1 expression is differentially regulated during organogenesis and participates in multimolecular nuclear condensates essential for the factor’s transcriptional activity. FOXN1’s C-terminal sequence regulates the diffusion velocity within these aggregates and modulates the binding to proximal gene regulatory regions. These dynamics are altered in a patient with a mutant FOXN1 that is modified in its C-terminal sequence. This mutant is transcriptionally inactive and acts as a dominant negative factor displacing wild-type FOXN1 from condensates and causing athymia and severe lymphopenia in heterozygotes. Expression of the mutated mouse ortholog selectively impairs mouse TEC differentiation, revealing a gene dose dependency for individual TEC subtypes. We have therefore identified the cause for a primary immunodeficiency disease and determined the mechanism by which this FOXN1 gain-of-function mutant mediates its dominant negative effect.

## Teaser

A dominant transcription factor mutation disrupting nuclear liquid phase separation causes human immunodeficiency.

## INTRODUCTION

The thymus microenvironment promotes the development of naïve T cells with a repertoire purged of “self” specificities and poised to react to potentially injurious “nonself” threats. Thymic epithelial cells (TEC) constitute the major component of the thymic stroma and can be categorized into separate lineages and states based on their specific molecular, structural, and functional characteristics (*1*). TEC differentiation, maintenance, and function critically rely on the transcription factor FOXN1 (*2*–*8*). FOXN1 is a member of the forkhead box (FOX) family of transcription factors and recognizes a 5-base pair (bp) consensus sequence (GACGC) via its centrally located DNA binding domain (the Forkhead domain, FKH) (*2*). Direct and water-mediated contacts via residues in an α helix inserted in the DNA major groove dictate the binding of the FKH to its consensus binding site (*9*). In addition, the first 154 N-terminal amino acids for FOXN1 are required for normal TEC differentiation, and the acidic activation domain in the C-terminal region is required for target gene transcription (*10*, *11*).

Many transcription factors operate within large nuclear biomolecular condensates, which resemble aggregates formed by liquid-liquid phase separation. The lack of a membrane surrounding nuclear condensates allows rapid exchange of components with the nucleoplasm (*12*). Nuclear condensates can affect chromatin architecture by maintaining the heterochromatin domain (*13*) or have been shown to drive gene activation through the assembly of transcriptional complexes at enhancer-rich gene regulatory regions (*14*). Proteins in membrane-less nuclear organelles characteristically include intrinsically disordered regions (IDRs) (*14*–*17*). FOXN1 has several IDRs, although whether or how it might function within a nuclear biomolecular condensate under physiological and pathological conditions has remained largely undefined.

Autosomal recessive mutations of *FOXN1* that result either in a premature stop in translation (p.R255X) (*5*, *18*), a loss of DNA binding (p.R320W) (*18*), or a frameshift and premature truncation (p.S188Afs*114) (*19*) give rise to a rare, phenotypically unvarying form of congenital severe combined immunodeficiency known as lymphoid cystic thymic dysgenesis (also knonwn as “nude” phenotype; ORPHA169095). In addition to the absence of thymic tissue and the consequent lack of T cells, the syndrome is further characterized by alopecia universalis and nail dystrophy as a result of a lack of functional FOXN1 expression in the ectoderm (*4*). In contrast to the complete constitutive absence of FOXN1 expression, reduced expression of FOXN1 in fetal and young mice causes only a transient hypoplasia of the thymus, (*20*) whereas lower FOXN1 concentrations in older animals are associated with premature thymic involution (*8*).

Together with recent clinical observations of individuals with heterozygous (*21*) or compound heterozygous (*22*) FOXN1 mutations, these findings suggest that the formation and maintenance of the thymus appear to be sensitive to small changes in FOXN1 availability, the consequences of which can be severe for the immune system. However, the molecular mechanism by which FOXN1 exerts its precise transcriptional function and whether this might involve nuclear biomolecular condensates has remained unclear (*23*, *24*).

## RESULTS

### Athymia and T cell lymphopenia caused by heterozygous FOXN1 mutants

**Fig. 1.**
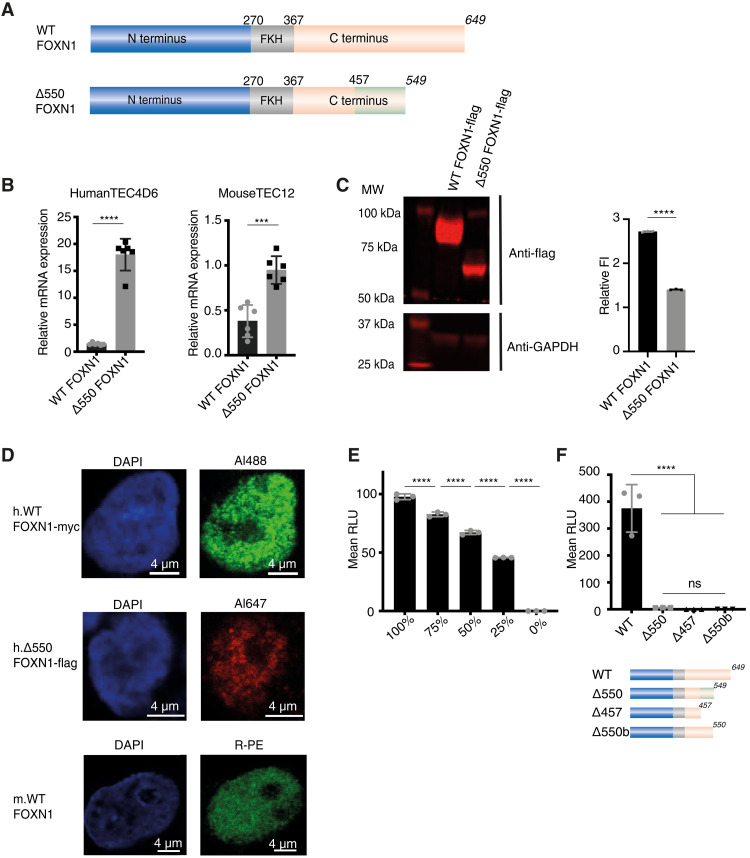
The Δ550 FOXN1 mutation lacks transcriptional activity. (**A**) Schematic representation of wild-type (WT) FOXN1 and the Δ550 FOXN1 mutant. FKH, forkhead domain. Numbers represent amino acids, numbers in italics indicate the position of the stop codon, and the green box indicates the scrambled amino acid sequence. (**B**) Relative wild-type and Δ550 FOXN1 mRNA expression compared to GAPDH in transfected human 4D6 and mouse TEC1.2 cells. (**C**) Western blot and quantification of FLAG-tagged wild-type and Δ550 FOXN1 protein in 4D6 cells. Fluorescence intensity (FI) was compared to that of GAPDH after background subtraction. (**D**) Confocal microscopy of the nuclear localization of untagged wild-type (bottom), myc-tagged wild-type, (middle), and FLAG-tagged Δ550 FOXN1 in 4D6 cells (top) using indirect and direct immunofluorescence, respectively, and DAPI counterstaining. (**E**) Linear relationship between wild-type FOXN1 gene dose and transcriptional activity in transfected 4D6 cells expressing a luciferase reporter construct. Luciferase activity was quantified as arbitrary relative light units (RLU). (**F**) Top: Transcriptional activity of wild-type and mutant FOXN1 in 4D6 cells as measured by luciferase reporter assay. Bottom: Schematic representation of FOXN1 variants with scrambled amino acid sequences shown as a light green box. The data are representative of one (D), two (C), three (E and F), and six (B) independent experiments. Each symbol represents data from biological (B and D) or technical (C, E, and F) replicates. Mean value and SD are shown and statistically compared by two-tailed unpaired *t* test (B, C, and F) and analysis of variance (E); ≥0.05 (ns), ***P < 0.001, and ****P < 0.0001.

We have identified a FOXN1 variant with a single base pair deletion in exon 7,

NM_001369369: c.1370delA (p.H457Pfs*93), in three individuals of a single kindred. This variant caused a frame shift resulting in a “scrambled” sequence of 92 amino acids and a premature stop codon at amino acid 549 of FOXN1 and is designated thereafter as Δ550 FOXN1 (Fig. 1A and fig. S1A). The affected individuals were heterozygous for the Δ550 FOXN1 mutation but presented clinically with athymia and T cell lymphopenia, the latter characterized in the index case by the absence of T cell receptor (TCR) excision circles (i.e., small circles of DNA created by rearrangement of TCR genes used as a surrogate marker to assess recent thymic T cell output) and a significantly reduced TCR repertoire diversity (fig. S1, B and C).

To characterize the Δ550 FOXN1 variant, we engineered a single base pair deletion at position 1370 in the human wild-type *FOXN1* sequence and added either a 3′ flag or myc tag to both the wild-type and mutant *FOXN1* genes. Wild-type and Δ550 *FOXN1* sequences were expressed using the same vector in human (TEC4D6) and mouse (TEC1.2) TEC lines. Higher mRNA levels were observed for the mutant compared to wild-type (Fig. 1B and fig. S1F), indicating that the Δ550 FOXN1 mRNA escaped nonsense mediated decay. In contrast, transfected TEC4D6 cells contained lower Δ550 FOXN1 protein levels when compared to the wild-type protein, suggesting that translation efficiency and/or stability were reduced for the mutant protein (Fig. 1C).

To determine Δ550 FOXN1’s subcellular localization, TEC4D6 cells were transfected with either wild-type or mutant FOXN1, and the location of each variant was determined by immunostaining. Both wild-type and Δ550 FOXN1 were present in the nucleplasm, expressed in a speckled pattern, and were excluded from the nucleolus, as was seen for untagged FOXN1 detected using an anti-FOXN1 antibody (Fig. 1D). The FOXN1 nuclear expression pattern is similar to transcription factors that are part of phase-separated multimolecular condensates (*14*).

The transcriptional activity of wild-type and Δ550 FOXN1 were assayed in TEC4D6 cells cotransfected with a reporter plasmid containing a luciferase gene under the transcriptional control of the FOXN1-specific *Psmb11* promoter, which normally controls β5t expression in TEC (*2*). Wild-type FOXN1 activated the luciferase gene in a gene dosage sensitive manner (Fig. 1E), whereas Δ550 FOXN1 failed to activate luciferase gene expression (Fig. 1F). Luciferase activity was also not observed in transfectants of C-terminally truncated wild-type FOXN1 variants, where a stop codon was introduced either at amino acid 457 (the position of the Δ550 FOXN1 missense mutation) or 550 (the Δ550 FOXN1 premature stop codon) (Fig. 1F), consistent with the C-terminal domain playing a critical role in FOXN1-mediated gene activation (*10*, *11*) . Although Δ550 FOXN1 apparently behaved as a loss-of-function mutant for transcriptional activation, its heterozygosity causing athymia was not explained by these results.

### Δ*505 Foxn1* heterozygous mice show TEC defects

**Fig. 2.**
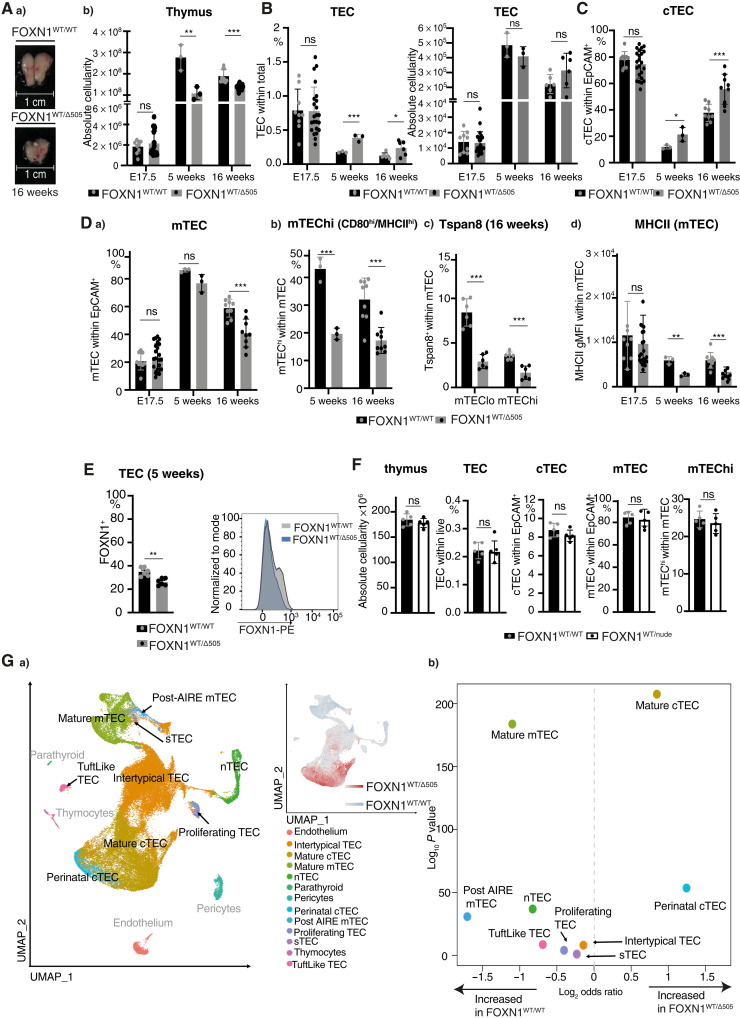
Heterozygous Δ505 FOXN1 expression in mice impairs TEC differentiation and promiscuous gene expression. Comparison of gender-matched FOXN1^WT/WT^ and FOXN1^WT/Δ505^ mice at indicated ages: (**A**) thymus gross anatomy and absolute cellularity. (**B**) Relative and absolute TEC cellularity. (**C**) Relative cTEC cellularity. (**D**) Relative cellularity of (a) total mTEC, (b and c) their phenotypically distinct subpopulations, and (d) level of MHCII expression on the surface of total mTEC. (**E**) Comparison of frequency of FOXN1^+^ cells and FOXN1 expression levels in TEC from gender-matched FOXN1^WT/WT^ and FOXN1^WT/Δ505^ mice at 5 weeks. (**F**) Cellularity of 5-week-old male FOXN1^WT/nude^ mice: total thymus cellularity and frequencies of total TEC, cTEC, mTEC, and mature (i.e., MHCH^high^) mTEC. (**G**) Single-cell TEC analysis of FOXN1^WT/WT^ and FOXN1^WT/Δ505^ mice. (a) Uniform Manifold Approximation and Projection (UMAP) analysis of combined single TEC transcriptomic data with an inset showing local enrichment of TEC single cells by genotype (using the 200 nearest neighbors). (b) Comparative analysis of TEC subtype frequencies. Each symbol in (A) to (F) represents data from an individual wild-type or mutant mouse. The flow cytometric gating strategies are in figs. S3 (A to D) and S9 (E). The data in (A) to (D) are from four (E17.5), one (week 5), and two to three (week 16) and for (E) from two independent experiments with each at least three wild-type and mutant male mice, for (F) from one independent experiment with five FOXN1^WT/WT^ and five FOXN1^WT/nude^ male mice. Mean value and SD are shown in bar graphs and were calculated two-tailed unpaired *t* test; ≥0.05 (ns), *P < 0.05, **P < 0.01, and ***P < 0.001. Data in (G) are from 18,637 and 36,943 cells isolated from three FOXN1^WT/WT^ and three FOXN1^WT/Δ505^ mice at 5 and 16 weeks of age. Data in (Gb) display log_2_ odds ratio and −log_10_ *P* values using Fisher’s exact test.

To analyze the impact of Δ550 FOXN1 heterozygosity on thymus development and function, mice were generated to have a single nucleotide deletion, orthologous to that observed in the index patient, at position 1370 of the murine *Foxn1* gene. The ensuing frame shift resulted in a scrambled protein sequence starting at amino acid 457 and a premature stop codon at amino acid 505 (named Δ505; fig. S2A). Five- and 16-week-old male mice (but not embryos) heterozygous for the Δ505 FOXN1 mutation (FOXN1^WT/Δ505^) had a thymus with significantly reduced overall cellularity when compared to age-matched wild-type littermates (FOXN1^WT/WT^) (Fig. 2A). While their total TEC counts were comparable with wild-type littermates (Fig. 2B), the postnatal FOXN1^WT/Δ505^ mice displayed changes in the relative number of flow cytometrically defined TEC subpopulations (Fig. 2, C and D). The relative frequencies of all TEC (EpCAM^+^CD45^−^) and that of cortical (c) TEC (EpCAM^+^Ly51^+^UEA^−^) were increased in FOXN1^WT/Δ505^ mice at both time points (Fig. 2, B and C). In addition to a lower frequency of total medullary (m) TEC (EpCAM^+^Ly51^−^UEA^+^) at 16 weeks, the differentiation within that lineage revealed a partial maturational block. The frequency of postnatal mature medullary (m) TEC (i.e., CD80^pos/hi^) was decreased (Fig. 2Db and fig. S2, D to E) with fewer CD80^pos/hi^ cells that expressed in an AIRE (autoimmune regulator)–dependent, promiscuous way the tissue-restricted antigen, Tspan8, and fewer cells that had progressed beyond that specific developmental stage (also known as post-AIRE, e.g., CD80^lo^ Tspan8^pos^; Fig. 2Dc). Moreover, mTEC and, in particular, cells with an immature phenotype expressed lower levels of major histocompatibility complex (MHC) class II on their cell surface (Fig. 2Dd and fig. S2F). However, the frequency of TEC expressing full-length FOXN1 and the geometric mean fluorescence intensity of wild-type FOXN1 in TEC were reduced in FOXN1^WT/Δ505^ mice when quantified using an antibody that binds the C terminus between amino acids 475 and 542 (Fig. 2E) (*25*). Both TEC frequency and cellularity were normal in age-matched FOXN1^WT/nu^ mice, i.e., mice heterozygous for the nude locus, which encodes a FOXN1 mutation unable to bind to DNA (Fig. 2F), suggesting that the differences observed were not secondary to a reduction in *Foxn1* gene dosage.

To further probe the consequences of Δ505 *Foxn1* heterozygosity for TEC differentiation, we assessed the transcriptome of single epithelial cells using a recently published dataset as reference to infer distinct TEC subtypes (*1*). The composition of the TEC scaffold was significantly changed in mutant mice, affecting the frequency of all but one of the TEC subtypes [i.e., structural (s)TEC; Fig. 2G] with concomitant alterations in biological pathways relating to antigen presentation, cytokine responses, and cell proliferation (tables S1 and S2). These changes in TEC lineage maturation included an expansion both of perinatal cortical (c) TEC, a subtype substantially reduced in wild-type mice 4 weeks of age and older, and mature cTEC. However, mature cortical (c) TEC had a significantly reduced expression of genes associated with epithelial cell proliferation (*Apoe*, *Gas1*, *Tacstd2*, *Xdh*, *Igf1*, *Sparc*, *Ccnd1*, *Trp63*, and *Fst*), suggesting likely deficits in the proliferative capacity in most TEC subtypes. Notably, mature mTEC from FOXN1^WT/Δ505^ mice showed a reduced expression of Myc pathway genes, previously demonstrated to be important for thymus size in senescence (*26*), relative to FOXN1^WT/WT^ mice (*P* = 7.531277 × 10^−06^). In contrast, the frequencies of almost all of the other TEC subtypes were reduced when compared to wild-type mice, with a particularly large reduction in mature and post-AIRE mTEC (Fig. 2Gb). The relative increase of TEC from the cortical rather than the medullary lineage in FOXN1^WT/Δ505^ mice was substantial among intertypical TEC, as we observed a significantly lower expression of *Krt5*, a marker of mTEC fate, and a higher expression of *Prss16*, a marker of cTEC fate (all *P* < 0.0001) in this composite subtype (fig. S2G). The expression of FOXN1 target genes was reduced in the mTEC compartment (fig. S2Ha) (*2*), which paralleled a reduced expression of AIRE-controlled tissue-restricted antigens (fig. S2Hb) (*27*).

Moreover, mature mTEC isolated from FOXN1^WT/Δ505^ mice at either 5 or 16 weeks of age showed evidence of accelerated aging in comparison to controls (*1*, *28*), thus suggesting that cells passing through the mTEC developmental block were under increased cellular stress (fig. S2Hc). In particular, mature mTEC from FOXN1^WT/Δ505^ mice showed increased expression of a gene associated with lysosomal injury (*Serpinba6*) but lower expression of a constituent of the immunoproteasome associated with alleviation of proteotoxic stress in mTEC (*Psmb10*) (*29*, *30*).

FOXN1^WT/Δ505^ mice displayed a normal coat, whereas mice homozygous for the Δ505 mutation (designated FOXN1^Δ505/Δ505^) were hairless and lacked a thymus and peripheral T cells (fig. S2I). These data are consistent with the clinical presentation of humans with the Δ505 mutation and previous studies showing that, although low levels of Foxn1 severely affect the thymus, they are still sufficient to generate a normal coat (*7*, *8*), whereas only the complete loss of functional FOXN1 impairs hair development.

**Fig. 3.**
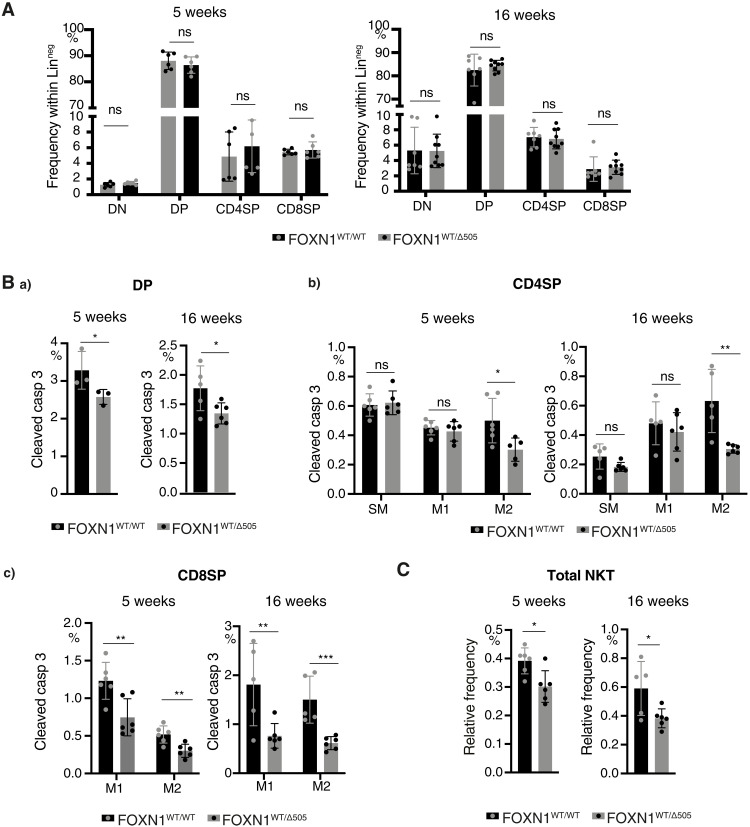
Heterozygous Δ505 FOXN1 expression impairs T cell clonal deletion at the population level. (**A**) Thymocyte differentiation in 5- and 16-week-old FOXN1^WT/WT^ and FOXN1^WT/Δ505^ mice. Live thymocytes negative for CD11b, CD11c, Gr1, CD19, CD49b, F4/80, NK1.1, TCRγδ, and Ter119 expression (designated Lin^neg^) were stained for the surface expression of CD4 and CD8. Gating strategy in fig. S5. (**B**) Age-specific analysis of the frequency of negatively selected (a) DP, (b) CD4 single positive (CD4SP) thymocytes, and (c) CD8 single positive (CD8SP) thymocytes as assessed by the detection of cleaved caspase 3 (casp 3). The CD4SP cells were differentiated into semimature (SM: CD69^+^MHC I^low^), mature 1 (M1: CD69^+^MHC I^+^), and mature 2 (M2: CD69^−^ MHC I^+^) thymocytes (*72*). The CD8SP cells are distinguished into mature 1 (M1: CD69^+^MHC I^+^) and mature 2 (M2: CD69^−^MHC I^+^) thymocytes. (**C**) Frequencies of NKT cells (TCRβ^+^, CD1d tetramer^+^) among thymocytes and (*73*) . Each symbol represents data from an individual wild-type or mutant mouse. Data are from three (A) and two (B and C) independent experiments, respectively. Mean value and SD are shown and statistically compared by two-tailed unpaired *t* test. ≥0.05 (ns), *P < 0.05, **P < 0.01, and ***P < 0.001.

We evaluated whether these epithelial changes affected thymopoiesis in FOXN1^WT/Δ505^ mice. At 5 and 16 weeks of age, thymocyte differentiation was generally comparable to that of wild-type mice (Fig. 3A and fig. S6). However, thymocyte negative selection was compromised in young and older FOXN1^WT/Δ505^ mice as fewer signaled (i.e., TCRβ^hi^) CD4^+^CD8^+^ [double positive (DP)] and mature stage 2 single CD4–positive cells (M2: CD69^−^ MHC I^+^) and single CD8–positive cells at the stages M1 (CD69^+^MHC I^+^) and M2 underwent clonal deletion (Fig. 3B). In addition, the frequency of natural killer T (NKT) cells (Fig. 3C) but not that of regulatory T cells (T_regs_) and γδ Τ cells was reduced in FOXN1^WT/Δ505^ mice (fig. S6, E and F). Thus, heterozygosity for a mouse ortholog of human Δ550 FOXN1 substantially impaired TEC differentiation and function.

### Δ550 FOXN1 is a dominant negative mutant

**Fig. 4.**
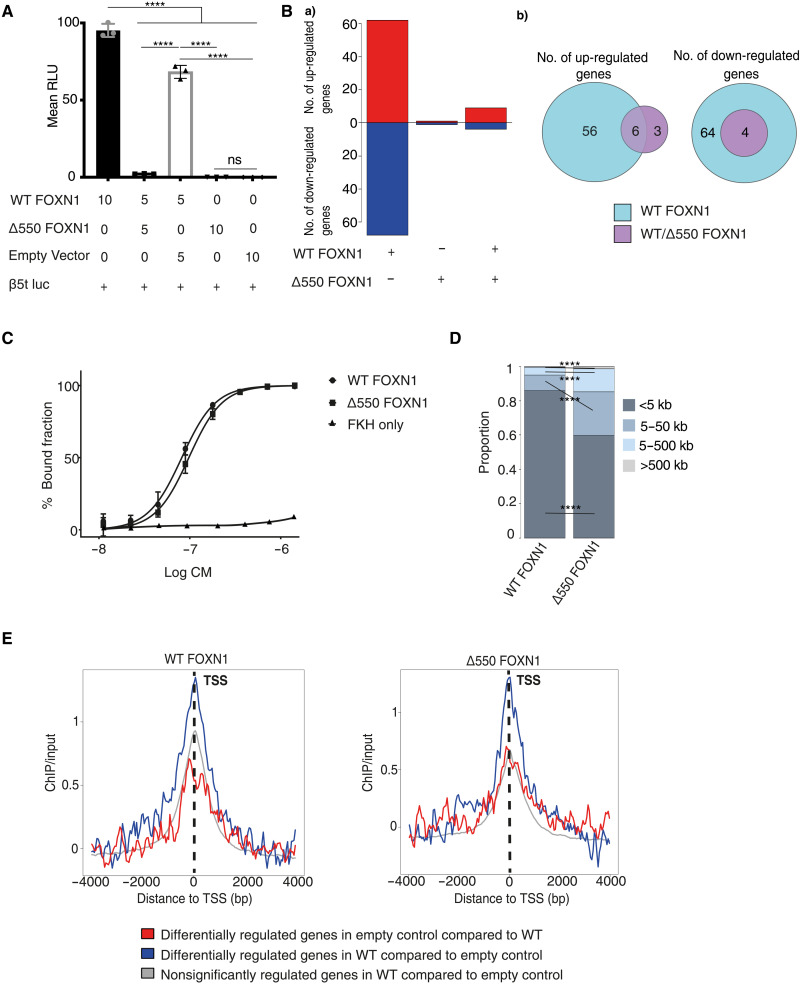
Δ550 FOXN1 is transcriptionally inactive and acts a dominant negative variant to compete with wild-type FOXN1 for DNA binding. (**A**) Expression of a luciferase reporter in 4D6 cells transfected with indicated expression vectors. Constitutive renilla expression was used in each transfectant as an internal control, and reporter activity was measured as arbitrary RLU following correction. (**B**) Gene expression changes in 4D6 cells transfected to express wild-type and Δ550 FOXN1 either alone or in combination; (a) number of genes differentially expressed in comparison to control transfected cells and (b) changes in the number of genes commonly or separately changed in the presence of wild-type and Δ550 FOXN1. (**C**) Gel shift assay to determine DNA binding affinity of wild-type FOXN1, Δ550 FOXN1, and isolated FOXN1 FKH to a DNA sequence containing the FOXN1-binding motif. Quantification of bound fraction fitted to a standard binding isotherm. (**D**) Proportion and comparison of distances to the closest transcriptional start site (TSS) for wild-type and Δ550 FOXN1 ChIP-seq peaks. (**E**) Wild-type (left) and Δ550 FOXN1 ChIP-seq peaks (right) flanking the TSS of genes differentially regulated by wild-type FOXN1. The data shown are representative of four (A) and three independent experiments (C), each with at least three technical replicates. (B), (D), and (E) display an independent experiment with three biological replicates for each sample. Mean value and SD are shown and were and statistically compared by two-tailed unpaired *t* test (A) and Fisher’s test (D): ns ≥ 0.05, *P < 0.05, **P <0.01, ***P < 0.001, and ****P < 0.0001.

Haploinsufficiency caused by hypomorphic *FOXN1* variants have been associated with T cell lymphopenia and thymic hypoplasia/aplasia (*21*) and thus may also explain the clinical phenotype of individuals heterozygous for the transcriptionally defective Δ550 FOXN1 variant (Fig. 1F). Alternatively, Δ550 FOXN1 could act as a dominant negative mutant interfering with the transcriptional activity of wild-type FOXN1. To test this hypothesis, we coexpressed wild-type and mutant Δ550 FOXN1 together with a FOXN1-specific luciferase reporter in TEC 4D6 cells (*2*). Contrary to the gene dose–dependent signal detected with wild-type FOXN1, coexpression of wild-type and variant FOXN1 substantially reduced luciferase activity (Fig. 4A), identifying Δ550 FOXN1 as a dominant negative mutant. Similar results were obtained in a murine TEC cell line coexpressing mouse wild-type FOXN1 and the Δ505 FOXN1 mutant (fig. S9A). Thus, a single nucleotide deletion generating the human Δ550 and mouse Δ505 mutations creates dominant negative variants of FOXN1.

To verify the dominant negative nature of Δ550 FOXN1, we analyzed gene expression profiles of the human TEC line 4D6 transfected with either wild-type or Δ550 FOXN1 alone or a combination of the two (Fig. 4B), using RNA sequencing (RNA-seq). Wild-type FOXN1 up-regulated 62 genes and down-regulated 68 genes. In contrast, Δ550 FOXN1 did not up-regulate any genes and down-regulated only a single gene, confirming its lack of transcriptional activity. Coexpression of wild-type and Δ550 FOXN1 resulted in the up- or down-regulation of only a fraction of genes controlled by wild-type FOXN1 [6 of 62 (9.6%) and 4 of 68 (5.8%), respectively] (Fig. 4B). The binding affinities of wild-type and Δ550 FOXN1 were statistically indistinguishable for a DNA probe containing two copies of the canonical GACGC motif (Fig. 4C) (*9*). Chromatin immunoprecipitation sequencing (ChIP-seq) analysis of 4D6 cells expressing either wild-type FOXN1-FLAG or Δ550 FOXN1-FLAG identified 10,273 and 16,426 peaks, respectively [at an irreproducible discovery rate (IDR) of <0.05] with significant enrichment in their overlap (122.1-fold, *P* < 0.0001; fig. S7A). FOXN1 binding occurred within a 5-kb window of the gene transcriptional start sites for 85.9% of sites bound by wild-type FOXN1 and 59.7% occupied by Δ550 FOXN1 (Fig. 4D). The consensus FOXN1-binding motif, GACGC (fig. S7B), was identified by the motif analysis tool MEME-ChIP (*31*) within wild-type FOXN1 ChIP-seq peaks as the top ranked candidate sequence, whereas the same motif was identified at a lower frequency in Δ550 FOXN1 ChIP-seq peaks (63.1% versus 49.2%, *P* < 0.0001). Hence, most wild-type but significantly fewer mutant FOXN1 molecules bound to proximal gene regulatory regions recognizing the same DNA motif (*P* < 0.0001), indicating that the C-terminal region of FOXN1 modulates both transactivation and DNA binding.

The FOXN1 ChIP-seq data were integrated with the RNA-seq data, revealing that only genes controlled by wild-type FOXN1 were also enriched for FOXN1 ChIP-seq signals in 4D6 cells (Fig. 4E). However, neither wild-type nor Δ550 peaks were enriched near the few genes differentially regulated by Δ550 FOXN1 (fig. S7C). *DLX3*, *GPC4*, and *CITED1*, which are all genes associated with epithelial or thymic development (*32*–*34*), were exclusively bound and transcriptionally controlled by only wild-type FOXN1. Together, these results support a mechanism by which Δ550 FOXN1 successfully competes with wild-type FOXN1 for DNA binding but because of its lack of a transactivation domain does not initiate transcription.

### Δ550 FOXN1 impairs condensate dynamics

**Fig. 5.**
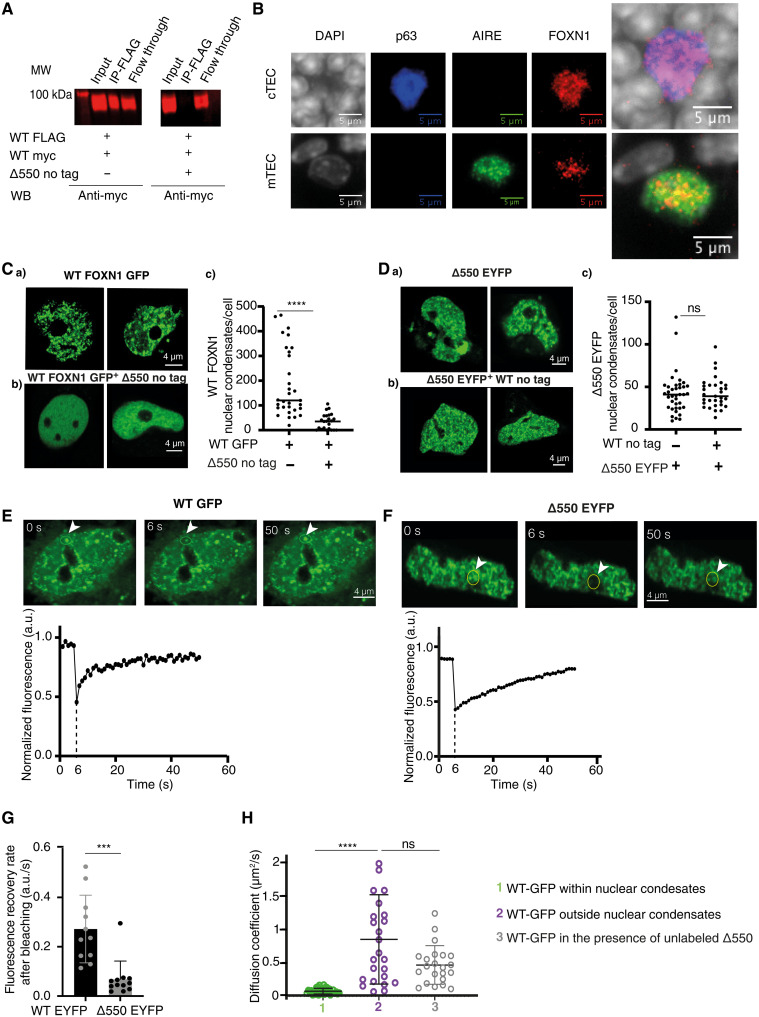
Δ550 FOXN1 disrupts the formation of wild-type FOXN1 multimers. Δ550 FOXN1 disrupts the formation of wild-type FOXN1 multimers. (**A**) Anti-Flag immunoprecipitation and Western blot (WB) analysis of FLAG- and myc-tagged wild-type FOXN1 expressed in the absence or presence of Δ550 FOXN1 in 4D6 cells. Protein detection using anti-myc antibody. (**B**) Widefield microscopy showing FOXN1 nuclear condensates in primary mouse TEC. FOXN1 (red), AIRE (green), and p63 (blue) direct immunofluorescence and DAPI counterstain of thymic tissue sections from 1-week-old wild-type mouse. AIRE-positive mTEC characteristically do not express p63. (**C**) Confocal microscopy showing nuclear condensates in 4D6 cells expressing (a) GFP-tagged wild-type FOXN1 either alone or (b) in the presence of untagged Δ550 FOXN1; (c) quantification of nuclear condensates per cell. (**D**) Confocal microscopy showing nuclear condensates in 4D6 cells expressing (a) EYFP-tagged Δ550 FOXN1 either alone or (b) in the presence of untagged wild-type FOXN1; (c) quantification of nuclear condensates per cell. (**E** to **G**) Fluorescence recovery after photobleaching (FRAP) analysis of transfected 4D6 cells. Top: photomicrographs of nuclei taken immediately before (0), 6, and 50 s after photobleaching of the indicated area (yellow circle). Bottom: fluorescence recovery curve of (E) GFP-labeled WT FOXN1 and (F) EYFP-labeled Δ550 FOXN1 as a function of time. a.u., arbitrary units. (G) Fluorescence’s recovery rate (a.u./s) of EYFP-labeled wild-type and Δ550 FOXN1 nuclear condensates following photobleaching. (**H**) FCS measurement of the diffusion coefficient (μm^2^/s) of GFP-tagged WT FOXN1 in the nucleus of 4D6 cells within and outside the nuclear condensates and in the presence or absence of untagged Δ550 FOXN1. Data shown are from three (A, C, D, E, F, and G), one (B), and one (H) independent experiments. Mean and SD are shown in (C), (D), (G), and (H), and the data were analyzed using the two-tailed unpaired *t* test (C, D, and G) and the Kolmogorov-Smirnov test (H). ns ≥ 0.05, ***P < 0.001, and ****P < 0.0001.

Transcription factors control gene expression as either monomers, homodimers, heterodimers, or oligomers (*35*). To investigate whether FOXN1 operates either as a dimer or a multimer, 4D6 cells were cotransfected with two forms of wild-type FOXN1, each labeled with either a FLAG- or myc-tag. Immunoprecipitation using an antibody directed against one tag also recovered FOXN1 labeled with the other tag, demonstrating the formation of FOXN1 homodimers or multimers. This complex formation was inhibited in the presence of the Δ550 variant (Fig. 5A), thus demonstrating the physical impact of the mutant on FOXN1 multimer formation. In contrast, immunoprecipitation of cell lysates of 4D6 cells cotransfected to express myc-tagged wild-type FOXN1 and FLAG-tagged Δ550 FOXN1 did not reveal a physical interaction between the two proteins (fig. S7D).

We investigated the nuclear behavior of wild-type and Δ550 FOXN1 expressed either alone or in combination. Live imaging of 4D6 cells expressing either wild-type or Δ550 FOXN1 (each labeled separately with a distinct fluorochrome) revealed large nuclear biomolecular condensates resembling aggregates formed by liquid-liquid phase separation and specialized in gene regulation and genome maintenance (Fig. 5, Ca and Da) (*36*–*41*). The FOXN1 nuclear condensates were also observed in wild-type TEC in thymus tissue sections (Fig. 5B), confirming their existence in primary TEC in situ. Coexpression of wild-type and Δ550 FOXN1 significantly decreased the number of condensates formed by wild-type FOXN1 (Fig. 5C, b and c), while those containing the Δ550 mutant remained unchanged (Fig. 5D, b and c). Thus, Δ550 FOXN1’s ability to interfere with the formation of nuclear condensates is consistent with its disruption of wild-type FOXN1 homomultimers.

Proteins in membrane-less nuclear organelles characteristically include IDRs, whose serine content is required for condensate formation (*14*–*17*). Both wild-type and Δ550 FOXN1 had IDRs with a higher serine content than ordered segments of the proteins (fig. S7, E and F). The lack of a membrane surrounding nuclear condensates allows rapid exchange of components with the nucleoplasm (*12*). We therefore used fluorescence recovery after photobleaching (FRAP) to study movement of fluorescently labeled FOXN1 in the condensates. Photobleaching of condensates in 4D6 cells expressing either wild-type or Δ550 FOXN1, followed by recovery for 50 s (Fig. 5, E and F), revealed that the Δ550 mutant had a reduced recovery rate (Fig. 5G), indicating that it only partially retains the dynamic properties of wild-type FOXN1. Moreover, the mutant displayed similarly a reduced recovery outside of nuclear condensates (fig. S5G), suggesting a generally impaired mobility. Similarly, FOXN1 variants truncated at either amino acid 457 or 550, and therefore, each with fewer IDRs formed nuclear condensates at a frequency comparable to that of wild-type or Δ550 FOXN1 (fig. S5H) but displayed decreased FRAP recovery rates (fig. S5I). Hence, different FOXN1 C-terminal mutants could form nuclear condensates, but shorter amino acid sequences significantly impaired exchange of FOXN1 between the condensates and the surrounding nucleoplasm.

As transcription factors translocate within the nucleoplasm and bind to their target sequence (*42*), they can form a range of interactions with other molecules that influence their behavior and function. Within biomolecular condensates, the transcription factor diffusion is slower compared to the rest of the nucleoplasm due to the organelles’ compact nature and the presence of other interacting molecules (*36*, *38*, *43*). Fluorescence correlation spectroscopy (FCS) revealed that diffusion of wild-type FOXN1 was substantially reduced within condensates compared to the adjacent nucleoplasm (11-fold, *P* < 0.0001; ≈0.07 μm^2^/s inside the condensate versus ≈0.8 μm^2^/s outside the condensate; Fig. 5H). Diffusion of wild-type FOXN1outside of condensates remained unchanged in the presence of Δ550 FOXN1 (≈0.5 μm^2^/s; Fig. 5H). These results are consistent with an exclusion of wild-type FOXN1 from the Δ550 FOXN1 condensates in which the FOXN1 dwell time is typically reduced and correlate with wild-type FOXN1’s reduced transcriptional activity in the presence of Δ550 FOXN1. Thus, wild-type FOXN1 is locally enriched in higher-order liquid-like aggregates, whose diffusion properties change in the presence of Δ550 FOXN1.

### Binding partners modulate FOXN1 transcription

**Fig. 6.**
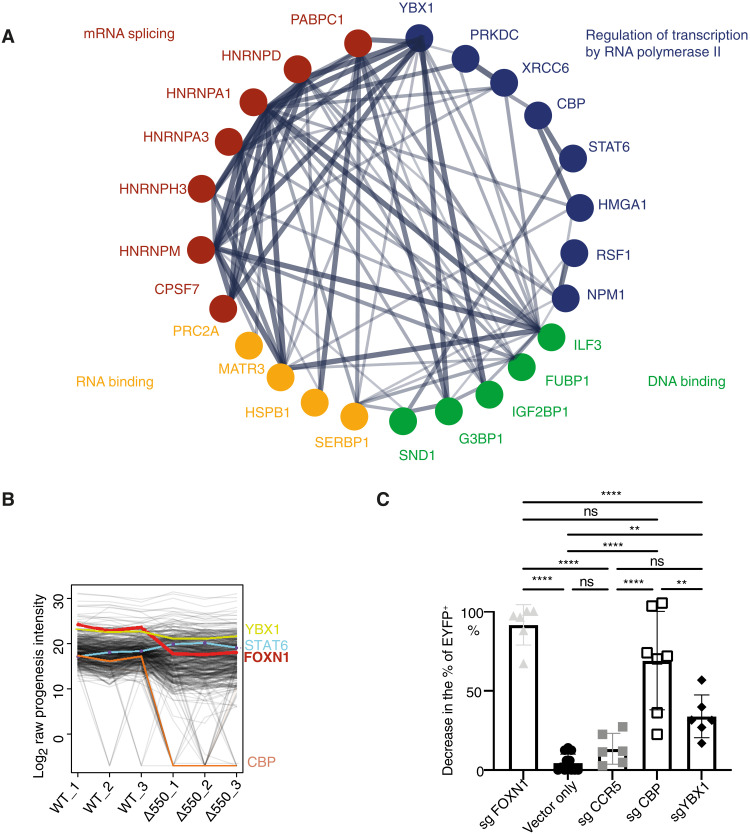
The FOXN1 interactome. (**A**) STRING protein-protein interaction analysis of FLAG-tagged wild-type and Δ550 FOXN1 pulldowns from transfected 4D6 cells and subsequent analysis by LC-MS/MS. The links show previously verified interactions whereby the thickness of individual lines represents the strength of experimental evidence for these interactions. (**B**) Normalized abundance of proteins identified by immunoprecipitation and (LC-MS/MS). Comparison of three samples of 4D6 cells each transfected to express wild-type and Δ550 FOXN1 showing the normalized abundance of FOXN1 (red line), STAT6 (blue line), CBP (orange line), and YBX1 (yellow line). (**C**) CBP and YBX1 are cofactors for FOXN1-mediated transcription. The frequency of cells positive for EYFP was measured using 4D6-EYFP^β5t^ FOXN1^CMV^ cells that had a CrispR-mediated deletion of either CBP, YBX1, FOXN1, or CCR5. 4D6-EYFP^β5t^ FOXN1^CMV^ cells transfected with only the cas9 vector served as an additional control. CrispR-targeted loss of FOXN1 and CCR5 served as a positive and negative control, respectively. The graph shows the relative decrease in the frequency of EYFP-positive cells after transfection with CBP, YBX1, FOXN1, and CCR5 sgRNAs. The frequency of EYFP-positive cells after transfection with the Cas9-mruby2 vector was set to 0 (no decrease in the % of EYFP-positive cells after transfection with the Cas9-mruby2 vector only), while the decrease in the % of EYFP-positive cells after transfection with the sgFOXN1 was set to 100. Any other changes were shown in relation to that. The data are representative for two (C) and one (A and B) independent experiments with three biological replicates each. Mean value and SD are shown and statistically compared by using a two-tailed unpaired *t* test; ns ≥ 0.05, **P < 0.001, and ****P < 0.0001.

Transcription factors interact with a large number of other molecules that modulate their function. To identify the FOXN1 interactome, we analyzed pulldowns from nuclear lysates of 4D6 cells expressing either wild-type or Δ550 FOXN1. Three hundred forty putative binding partners were identified by liquid chromatography with tandem mass spectrometry (LC-MS/MS). To exclude false positives, we required candidate proteins (i) to be detected in all replicates of wild-type or Δ550 FOXN1 precipitates; (ii) to not correspond to known contaminants (e.g., albumin, keratins, histones, cytoplasmic proteins, and ribosomal proteins) (*44*); and (iii) to localize to the nucleus according to the UniProt Knowledgebase (*45*). The number of candidates was further reduced to 32 (Fig. 6A), applying two additional suppositions. First, we considered a situation where the quantity of binding partners that recovered from precipitates would correlate with the availability of FOXN1. Because of the higher protein amount of wild-type FOXN1 compared to that of the mutant in transfected 4D6 cells (Fig. 1C and fig. S8A), the amount of candidate proteins was expected to be higher in precipitates of wild-type FOXN1 [as shown for Y box–binding protein 1 (YBX1) and Creb binding protein (CBP) in Fig. 6B]. We also considered a scenario where a protein partner characteristically associated with wild-type FOXN1 would be sequestered away by Δ550 FOXN1 as a result of the mutant’s higher affinity for the binding partner. Here, the amount of the candidate binding partner present in immunoprecipitations would directly correlate with the presence of Δ550 FOXN1 and inversely compare to the concentration associated with wild-type FOXN1 [signal transducer and activator of transcription 6 (STAT6) in Fig. 6B]. Of the 32 candidate FOXN1 binding partners, 30 behaved in accordance with the first assumption, and 2 behaved as expected for the second supposition (table S3). These FOXN1 interactome candidates included (i) mRNA splicing factors, (ii) transcriptional coactivators, (iii) DNA binding, and/or (iv) RNA binding proteins (Fig. 6A).

Selected interactome candidates were tested for their role in FOXN1-controlled gene transcription using 4D6 cells stably transduced with a lentiviral vector constitutively expressing FOXN1 and containing a minimal *Psmb11* promoter (*2*) controlling the expression of enhanced yellow fluorescent protein (EYFP) (designated 4D6-EYFP^β5t^ FOXN1^CMV^ cells; fig. S8, C and D). Changes in EYFP levels in the reporter cells reflect changes in FOXN1 functionality. We identified the transcriptional coactivator CBP and RNA binding protein YBX1 as potential FOXN1 interaction partners. Both proteins correlated positively with the amount of immunopreciptated FOXN1 protein, contained several IDRs and a higher disordered confidence score (Fig. 6B and fig. S7F), and could be detected in all TEC subtypes (*1*). Endogenous CBP also colocalized in transfected 4D6 cells with FOXN1 (Pearson correlation, 0.54) (fig. S8B). Deletion of *Cbp* and *Ybx1* resulted in a significant reduction of FOXN1-mediated activation of EYFP in 4D6-EYFP^β5t^FOXN1^CMV^ cells (Fig. 6C), demonstrating that both proteins play a role in FOXN1-regulated gene expression.

### FOXN1 expression is developmentally regulated

**Fig. 7.**
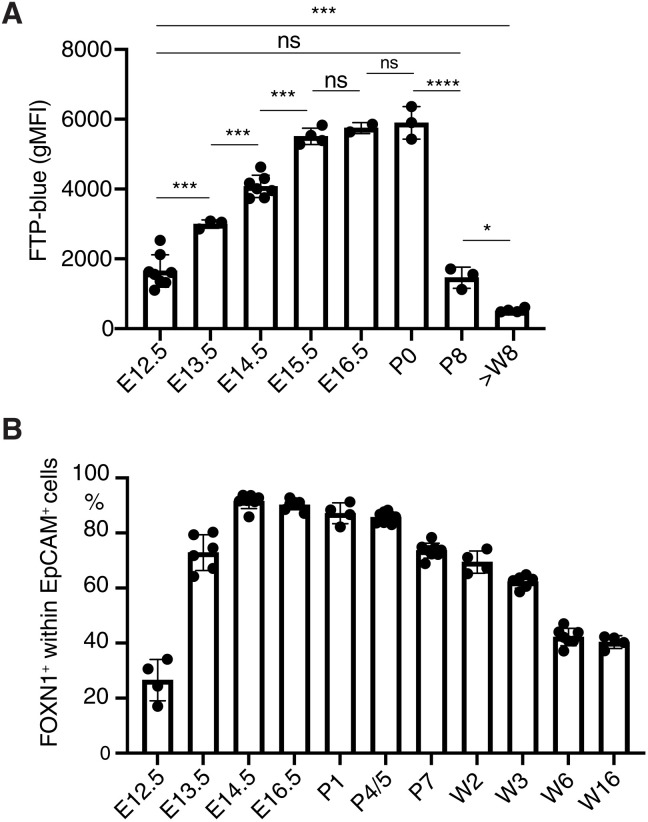
*FOXN1* promoter activity is developmentally regulated. (**A**) Geometric mean fluorescent intensity (gMFI) of FTP at 465 nm (FTP-blue) in total TEC from FTP^FOXN1^mice. (**B**) Frequency of FOXN1^+^ cells in total TEC from FOXN1^WT/WT^ mice at indicated ages. Each dot represents an individual mouse. The data are from two independent experiments at each time point with at least three mice each. Mean value and SD are shown and were calculated with two-tailed unpaired *t* test ≥0.05 (ns), *P < 0.05, ***P < 0.001, and ****P < 0.0001.

**Fig. 8.**
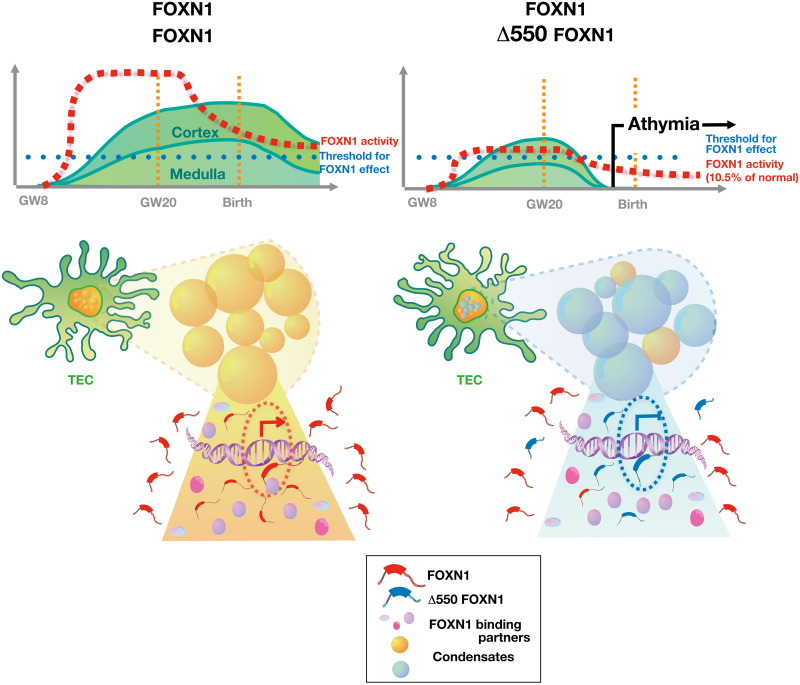
Graphical model explaining how Δ550 FOXN1 compromises thymus tissue maintenance. Nuclear FOXN1 (red symbols) concentration is required to be above a critical limit (blue dotted line) to initiate and maintain TEC differentiation. Owing to its dynamic promoter activity, the level of FOXN1 (red broken line) greatly exceeds this threshold during the second trimester and remains sufficiently elevated for an extended period of time thereafter. This allows FOXN1 to accumulate in sufficient quantities in nuclear condensates (yellow spheres), interact there with essential binding partners, and drive the transcription of target genes (left). In individuals, heterozygous for the Δ550 FOXN1 mutant (blue symbols), wild-type FOXN1 is however expressed from a single allele, and its concentration is thus reduced (right). Furthermore, wild-type FOXN1 molecules are competitively displaced by Δ550 FOXN1 from condensates so that most of these structures contain mostly the transcriptionally inactive variant (blue spheres). After the second trimester, transcription of the *FOXN1* locus is reduced, and consequently, only few condensates retain wild-type FOXN1–controlled transcriptional activity, which collectively are insufficient to secure FOXN1 activity above its critical threshold. As a consequence, TEC are not maintained, and functional thymus tissue is lost causing athymia with peripheral T lymphopenia.

To understand the mechanism by which heterozygosity for Δ*550 FOXN1* and Δ*505 FOXN1* could initially support thymus organogenesis but over time give way to thymic aplasia or hypoplasia, we reasoned that given the dominant negative effect of these mutants was incomplete (Fig. 4, A and B, and fig. S9A), sufficient wild-type FOXN1 activity could be generated from a highly active *FOXN1* promoter during the early phases of thymus organogenesis. To test this idea, we engineered mice that expressed a fluorescent timer protein (FTP) under the control of the *FOXN1* promoter. The FTP initially emits a blue fluorescence (peak at 465 nm) but progressively matures within 24 hours to emit a red fluorescence (604 nm; fig. S9B) (*46*). The FTP’s geometric mean fluorescent intensity (gMFI) at 465 nm correlated with *FOXN1* promoter strength. gFMI gradually increased from embryonic day 12.5 (E12.5), reaching a plateau 4 days later until birth, when it rapidly diminished to values below those observed at early embryonic time points (Fig. 7A and fig. S9C). These dynamic shifts were paralleled by changes in the frequency of FOXN1-positive cells (Fig. 7B). Thus, the activity of the FOXN1 promoter varies significantly over time. This, together with the observation that heterozygosity for the Δ505 allele diminished FOXN1 protein levels in TEC, supports our hypothesis that residual mouse FOXN1 activity is insufficient for normal thymus function in FOXN1^WT/Δ505^ mice after birth. Specifically, we estimated the residual FOXN1 activity in the presence of the mutant. We took as a measure of FOXN1 activity the log fold change of those genes significantly up-regulated by wild-type FOXN1 in 4D6 cells transfected by wild-type FOXN1 versus nontransfected cells. By estimating the log fold change of those genes in cells cotransfected with wild type and mutant, the effective wild-type function in the presence of the mutant was found to be 10.5% of that under physiological conditions (Fig. 8).

## DISCUSSION

FOXN1 is a gene dosage–sensitive transcription factor required for the differentiation and function of thymic epithelia. Mutations in FOXN1 have so far been linked to a recessive loss of function and, more recently, to hypomorphic variants believed to cause pathology through reduced gene dosage (*4*, *8*, *10*, *21*, *47*). Individuals heterozygous for *FOXN1* mutations that alter the protein’s C terminus are characteristically T cell lymphopenic (primarily affecting CD8 T cells) and, when symptomatic, display various pathologies including severe recurrent infections (*21*, *22*, *47*). Here, we describe a spontaneous *FOXN1* variant and elucidate the mechanism by which this and other C-terminal mutants exert a dominant negative effect on wild-type FOXN1.

Our data reveal that wild-type FOXN1 forms multimers that interact with other nuclear factors to form condensates poised to initiate transcription. FOXN1 condensate formation and DNA binding via its FKH domain require sequences in the C-terminal domain, whereas the N-terminal domain is dispensable for DNA binding (*10*). Nuclear condensates mediate transcription by concentrating and promoting efficient molecular interactions within a discrete biochemical milieu (*36*–*39*). Immunoprecipitations with wild-type FOXN1 identified several putative interacting partners commonly detected in these nuclear structures and collectively involved in mRNA splicing, transcriptional regulation, and DNA/RNA binding (*36*, *38*, *48*, *49*). One such partner is the transcriptional coactivator CBP, which serves as a central node in eukaryotic transcriptional networks by interacting via its IDRs with other proteins (*17*, *50*). IDRs, which are also present in FOXN1 and YBX1, use a small number of residues (*16*) or their posttranslational modifications (*17*) to promote high-specificity, modest-affinity interactions (*51*, *52*). The role of FOXN1’s interaction with CBP and, to a lesser extent, YBXI is demonstrated by the requirement of both cofactors to initiate *Psmb11* promoter–controlled gene transcription.

The shortened Δ550 FOXN1 with its partially altered C-terminal sequence preserves three of the six IDRs present in wild-type FOXN1 and retains the capacity to form condensates. Although IDRs have been suggested to enable phase separation, this capacity may, alternatively, be controlled by sequences with negative charges, high number of aromatic/hydrophobic residues, and/or a significant serine bias (*14*, *16*). Independent of the precise mechanism in play, the difference in sequence between wild-type and mutant FOXN1 demonstrates that the three terminal IDRs are not required for condensate formation. Instead, changes to the C-terminal sequence correlate with increased residence, separating condensate formation from dwell time.

Our in vitro experiments indicate that the DNA sequences bound by Δ550 FOXN1 are in part different compared to those bound by wild-type FOXN1, suggesting that the C-terminal sequences influence FOXN1 interactions with proximal gene regulatory regions. Nuclear condensate formation by the mutant FOXN1 is, however, unimpaired, indicating that condensate formation does not require CBP binding, although CBP is required for wild-type FOXN1 transcriptional activity.

The changes in the C-terminal sequence increase Δ550 FOXN1’s dwell time within nuclear condensates and result in a slower recovery rate in the nucleoplasm outside of these structures, suggesting an overall impaired mobility when compared to wild-type FOXN1. Thus, in cells expressing both wild-type and Δ550 FOXN1, the latter preferentially accumulates in the condensates due to its longer residence time, displacing wild-type FOXN1 and precluding it from activating transcription. It is also possible that the lack of CBP binding to Δ550 FOXN1 impairs normal displacement of the transcriptionally inactive interactome from DNA binding, further blocking access of wild-type FOXN1 to its target sequences. Functional FOXN1 is not completely excluded from nuclear condensates in the presence of Δ550 FOXN1, as limited gene transcription by wild-type FOXN1 is seen under coexpression conditions.

The heterozygous expression of Δ505 *Foxn1* in mice, the ortholog of the human mutation, results in thymic hypoplasia and changes in the composition of the TEC scaffold. The frequency and absolute cellularity of cortical epithelia are increased in FOXN1^WT/Δ505^ mice, whereas in wild-type mice, perinatal cTEC represent approximately one-third of all thymic epithelia at week 1 of age but, 3 weeks later, only contribute less than 1% of the epithelial scaffold (*1*). The differentiation of perinatal cTEC to mature epithelia is, however, unaffected in the presence of Δ505 FOXN1, consistent with the idea that a decrease in the normal FOXN1 gene dosage still allows for the maintenance of perinatal cTEC and is sufficient for their progression to mature cortical TEC. These effects are less marked than those caused by Δ550 FOXN1 in patients. This difference parallels the finding that the corresponding mouse variant is also less potent in vitro in impeding the transcriptional activity of its wild-type counterpart when compared to the human mutant and assessed by luciferase assay. The more subtle thymic phenotype in mice compared to that of human patients along with the lower potency of the mouse mutant in vitro are likely to be due to differences in the biological context and possibly species disparities. In support of this, murine thymopoiesis actively contributes to the peripheral lymphocyte compartment throughout life in contrast to human thymopoiesis (*53*), which may explain why the orthologous Δ505 FOXN1 mouse model does not completely recapitulate the degree of athymia seen in human patients.

TEC cellularity is unchanged in FOXN1^WT/Δ505^ animals despite thymic hypoplasia, underscoring that the functional competence of the TEC scaffold is compromised by the expression of the mutant FOXN1 and the consequent changes in the frequency of individual TEC subtypes. The frequency of thymocytes undergoing programmed cell death is reduced among CD4^+^CD8^+^ cells, indicating a qualitative deficiency of mutant cTEC when compared to wild-type epithelia. In contrast to cTEC, almost all other TEC subtypes in FOXN1^WT/Δ505^ animals are reduced in their frequency. Moreover, mTEC display a partial block in lineage maturation, reduced MHC class II expression, and impaired expression of peripheral tissue specific antigens, a phenomenon known as promiscuous gene expression and important to establish central T cell tolerance (*27*). These changes parallel a reduction in late maturational stages of postselection thymocytes. Moreover, the frequency of NKT cells, but not that of T_regs_, is reduced in FOXN1^WT/Δ505^ mice. As both lymphoid lineages depend on specific TEC niches, differential changes in the epithelial scaffold of FOXN1^WT/Δ505^ mice must account for this variance, such as tuft-like TEC and mTEC^lo^, which critically support NKT cell development (*54*). This conclusion is consistent with the expression of a dominant negative form of FOXN1 affecting TEC maturation differently depending on the cells’ lineage and developmental stage.

Last, our study indicates why individuals heterozygous for the Δ550 FOXN1 mutation are athymic at birth and yet were able to produce a limited population of peripheral T cells. Our data suggest that physiological changes in *FOXN1* promoter activity account for the observed loss of thymic tissue during the later stages of gestation (Fig. 8). Early in development, high levels of wild-type FOXN1 are generated because of the high activity of the *FOXN1* promoter, and this provides sufficient wild-type protein to mediate transcription despite the presence of the dominant negative mutant variant. This balance in favor of wild-type FOXN1 is further helped by the mRNA-encoding Δ550 FOXN1 being less efficiently translated when compared to wild-type transcripts. With increasing age, *FOXN1* promoter activity decreases, and the availability of wild-type FOXN1 protein is reduced to a nuclear concentration below the critical threshold required to maintain TEC function (*2*, *8*). The exact time when thymopoiesis stops in Δ*550 FOXN1* heterozygous patients can be estimated to have happened after week 20 of gestation because *N*-nucleotide additions to the TCR β chain’s CDR3 are similar for the index patient and healthy individuals, arguing for a time point when the completion of thymus organogenesis and the expression of terminal deoxynucleotidyl transferase has already occurred (*55*) (*56*). In the Δ505 FOXN1 mouse model, this time point corresponds to the first days after birth (*57*) and is consistent with thymus hypoplasia being observed in these animals only after birth.

Together, we identify a critical mechanistic determinant of thymic development by demonstrating that FOXN1 participates in the formation of nuclear condensates essential for transcription. Moreover, we provide a molecular explanation how a previously unidentified FOXN1 mutant dominantly interferes with the dynamic formation of transcriptional hubs ultimately causing a form of a primary immunodeficiency. The concept that liquid-liquid phase separation in biomolecular condensates helps compartmentalize distinct cellular processes has recently been discerned. Here, we demonstrate that the presence of a key developmental transcription factor within nuclear condensates correlates with its downstream function and that spontaneously occurring variants of this factor disrupt condensate formation.

## MATERIALS AND METHODS

### Patient recruitment

The patient was recruited as part of the OxClinWGS programme to apply clinical-grade whole-genome sequencing for patients with a broad range of rare diseases to identify the pathogenic mutations. Written informed consent was obtained from the patient as part of a study approved by the South Central Research Ethics Committee reference REC 12/SC/0044.

### Whole-genome sequencing analysis pipeline

DNA was extracted from peripheral blood mononuclear cells (PBMCs) of the father, mother, sibling, and the index patient using a magnetic bead method and the QIAsymphony automated extraction system (Qiagen). TruSeq polymerase chain reaction (PCR)–free libraries were prepared for all samples. Genome sequencing was performed with the HiSeq2500 (Illumina) instrument at the clinically accredited Molecular Diagnostics Laboratory at the John Radcliffe Hospital. Paired 100-bp reads were mapped to hs37d5 using BWA v.0.7.10 and Stampy v1.0.23 (www.well.ox.ac.uk/stampy). Samtools v1.3 was used to sort and merge the BAM files, and Picard markduplicates v1.111 was used to remove duplicates from the BAM files. The mean read depth was 30×. Variant calling was performed with Platypus (www.well.ox.ac.uk/platypus) using default settings except for minFlank = 0.

Variants were filtered for those with a call quality of at least 20 that passed upstream pipeline filtering. Variants were also required to have an allele fraction at least 5% and lie outside the top 5% most exonically variable 100-bp windows in healthy public genomes (1000 genomes). Variants also had to lie outside the top 1% most exonically variable genes in healthy public genomes (1000 genomes). We then excluded variants that are observed with a population allele frequency of ≥0.1% in the 1000 genomes project, ≥0.1% in the National Heart, Lung, and Blood Institute exome sequencing project (NHLBI ESP) exomes (all), ≥0.1% in the allele frequency community (AFC), ≥0.1% in exome aggregation consortium (ExAC) (maximum), or ≥0.1% in the genome aggregation database (gnomAD) (maximum). We then kept exonic variants (up to 20 bp into the intron) that are frameshifts, in-frame indels, stop codon changes, missense, or that splice site-altering variants (within 8 bp into intron or predicted to disrupt splicing by MaxEntScan). Last, we filtered for variants that were heterozygous in all three affected family members and were not seen in the unaffected mother.

Variant filtering was performed using QIAGEN Clinical Insight Interpret version 7.1.20210316 (https://variants.ingenuity.com/qci), and data were exported on 17 March 2021. The content versions of databases used in this commercial software are as follows: CADD (v1.6), Allele Frequency Community (2019-09-25), EVS (ESP6500SI-V2), Refseq Gene Model (2020-04-06), JASPAR (2013-11), Ingenuity Knowledge Base Snapshot Timestamp (2021-03-02 01:49:28.442), Vista Enhancer (2012-07), Clinical Trials (B-release), MITOMAP: A Human Mitochondrial Genome Database. www.mitomap.org, 2019 (2020-06-19), PolyPhen-2 (v2.2.2), 1000 Genome Frequency (phase3v5b), ExAC (0.3.1), iva (Nov 20), TargetScan (7.2), phyloP (GRCh37/hg19, 2014-02), GENCODE (Release 33), CentoMD (5.3), Ingenuity Knowledge Base (B-release), OMIM (July 06, 2020), gnomAD (2.1.1), BSIFT (2016-02-23), TCGA (2013-09-05), ClinVar (2020-09-15), DGV (2016-05-15), HGMD (2020.4), dbSNP153 (GRCh37/hg19), and SIFT4G (2016-02-23).

### Cell culture and transfection

The 4D6 cells (*58*) were grown in RPMI 1640 (Sigma-Aldrich) supplemented with 10% (v/v) heat-inactivated fetal bovine serum (FBS) (Scientific Laboratory Supplies Ltd.), 10 mM Hepes buffer, 1% (v/v) nonessential amino acids (NEAA) (Lonza), 2 mM l-glutamine (Lonza), penicillin (100 U/ml), and streptomycin (100 μg/ml; Lonza) at 37°C with 5% CO_2_ in a humidified incubator. Murine TEC1.2 cells were grown in Iscove’s modified Dulbecco’s medium (Life Technologies) supplemented with 2% (v/v) heat-inactivated FBS (SLS), 38.6 mM NaHCO_3_ (Sigma-Aldrich), 50 mM 2-Mercaptoethanol (Sigma-Aldrich), and Primatone RL/UF (0.3 g/liter; Fluka) at 37°C with 10% CO_2_ in a humidified incubator. Cells were seeded in cell culture plates of various sizes (Corning) and allowed to grow to reach 70% confluency by the time of transfection. Within 18 to 20 hours after seeding, cells were transfected with plasmids of interest using Fugene (Promega) as per the manufacturer’s recommendations. Four hours after transfection, the cell culture medium was replaced by fresh media. Cells were harvested 48 hours after transfection for further analysis.

For the assessment of the FOXN1 mRNA levels by quantitative reverse transcription PCR (qRT-PCR), 4D6 or TEC1.2 cells were transfected with plasmids encoding either wild-type-FOXN1 or Δ*550*-FOXN1 mutant along with a green fluorescence protein (GFP)–expressing vector in a 4:1 ratio to facilitate sorting of positively transfected cells. The wild-type and Δ550 FOXN1 mRNA levels were assessed in the fluorescence-activated cell sorting (FACS)–sorted cells.

### Plasmid construction

Human FOXN1 cDNA was purchased from Genecopoeia (sequence accession number BC140423). Full-length FOXN1 cDNA without a stop codon was cloned into vector backbones positioning at its C terminus either a flag (pCSF107mT-GATEWAY-3′-FLAG, Addgene), myc (pCSF107mT-GATEWAY-3′-Myc tag, Addgene), GFP, or an EYFP sequence (pcDNA1.3^−^, Addgene) using standard cloning methods. Mouse wild-type FOXN1 was cloned in a pLKP1 vector and Δ550 FOXN1 flag in a MR207953 vector using standard cloning methods. Insertion of single base pair changes for the generation of human and mouse FOXN1 variants was achieved by site-directed mutagenesis using the Phusion site-directed mutagenesis kit (Thermo Fisher Scientific). Specific primers for the introduction of single base pair deletions or single base pair changes were designed using the following software: www.agilent.com/store/primerDesignProgram.jsp. All plasmids were verified by Sanger sequencing.

### RNA, cDNA synthesis, and qRT-PCR

RNA isolation was performed using the Rneasy Mini (Qiagen) or Rneasy Plus Micro kit (Qiagen). cDNA was synthesized using a SensiFAST cDNA kit (Bioline) and assessed by qPCR (SensiMix SYBR Hi-Rox, Bioline). Expression levels of each gene were normalized against *GAPDH* expression, and fold change was calculated using the *2^-ΔCT^* or *2^-ΔΔCT^* equations (*59*). The following primer sequences were used: human *GAPDH*, 5′-AACAGCCTCAAGATCATCAGC-3 (forward) and 5′-CTGTTGCTGTAGCCAAATTCG-3′ (reverse); human *FOXN1*, 5′-TTCCTTACTTCAAGACAGCAC-3′ (forward) and 5′-GGTTCTTGCCAGGAATGG-3′ (reverse); mouse *Gapdh*, 5′GGTGAAGGTCGGTGTGAACG3′ (forward) and 5′ACCATGTAGTTGAGGTCAATGAAGG3′ (reverse); mouse *Foxn1*, 5′TCTACAATTTCATGACGGAGC3′ (forward) and 5′TCCACCTTCTCAAAGCACTTGTT3′ (reverse).

### Luciferase reporter assay

The luciferase reporter assay was performed as described previously (*2*). In short, 4D6 cells were cotransfected with a renilla control plasmid (pRL Promega) and a luciferase reporter plasmid (pGL4.10[Luc2], Promega) under the control of a minimal wild-type *Psmb11* promoter (designated β5t-luc) or a β5t promoter with a mutated FOXN1-binding sites (β5t-mut-luc) plus a FOXN1 construct of interest in a ratio of 1:10:10. Luciferease activity was measured 24 hours later using the Dual-Lucifearase Assay kit (Promega).

### Protein preparations and Western blot

For Western blot analysis, 4D6 were treated with lysis buffer [25 mM tris-HCL (pH7.5), 50 mM NaCl (Sigma-Aldrich), 0.1% NP-40 (Sigma-Aldrich), 0.1% SDS (Sigma-Aldrich), 0.5% sodium deoxycholate (Sigma-Aldrich), 10% glycerol (Sigma-Aldrich), and protease inhibitors (1 tablet/10 ml; Roche)] followed by SDS–polyacrylamide gel electrophoresis and immunoblotting, using mouse anti-FLAG (monoclonal M2, Merck), rabbit anti-myc (Cell Signaling Technology), and rabbit anti–glyceraldehyde phosphate dehydrogenase (GAPDH) (Cell Signaling Technology) antibodies. For the relative quantification of FOXN1 in Fig. 1C, the loading control was run through the gel from the same loading well as the experimental sample. After the protein transfer, the membrane was cut in two parts at about 50 kDa, and each part was developed separately with anti-FLAG (top part) and anti-GAPDH (bottom part). For the relative quantification of FOXN1 protein levels, the fluorescence intensity of WT or Δ550 mutant FOXN1 protein bands were calculated relative to the fluorescence intensity of the GAPDH loading control after subtracting background fluorescence.

### Immunofluorescence

The 4D6 cells were plated into 12-well cell culture plates containing sterile coverslips coated with lysine [1:10 dilution in phosphate-buffered saline (PBS), Sigma-Aldrich] and transfected as described earlier. For the immunofluorescent staining, cells were fixed with 4% paraformaldehyde solution, permeabilized with 0.2% Triton X-100, blocked in blocking buffer [1× PBS, 1% bovine serum albumin, and 5% goat serum (all Sigma-Aldrich reagents)], and subsequently stained with primary antibodies mouse anti-FLAG (1:500) (monoclonal M2, Merck) or rabbit anti-myc (1:200) (monoclonal, Cell Signaling Technology) and secondary antibodies [goat anti-rabbit Alexa Fluor 488 and goat anti-mouse Alexa Fluor 647,(Sigma-Aldrich)]. Where indicated, 4D6 cells were counterstained with 4′,6-diamidino-2-phenylindole (DAPI) (10 pg/ml in methanol), and all slides were mounted with ProLong Gold Antifade Mountant (Thermo Fisher Scientific). A ZeissLSM 880 inverted confocal laser scanning microscope was used to visualize the stained cells.

Frozen tissue sections were cut at 7-μm thickness, fixed with 3.6% formalin, permeabilized with 0.2% Triton X-100, and stained with R-Phycoerythrin–labeled (Abcam) anti-FOXN1 (a gift of H. R. Rodewald) (*25*), anti-deltaNp63 (BioLegend), and anti-AIRE (Invitrogen) antibodies. Unlabeled primary antibodies were detected using secondary antibodies (goat anti-rat Alexa Fluor 488 or goat anti-rabbit Alexa Fluor 647, both from Thermo Fisher Scientific). Tissue was counterstained with DAPI (10 pg/ml final dilution in PBS) and mounted with Hydromount Histology mounting media (National Diagnostics). Images were acquired using a Leica DMi8 microscope and analyzed by Fiji (ImageJ software).

### RNA sequencing

The 4D6 cells were transfected with plasmids encoding (i) wild-type-FOXN1 alone, (ii) Δ*550*-FOXN1 mutant alone, (iii) wild-type and Δ550, οr (iv) empty vector only, along with a GFP-expressing vector in a 4:1 ratio to facilitate sorting of positively transfected cells. Three biological replicates were prepared for each sample. A total of 75,000 GFP-positive cells were FACS-sorted, and RNA was extracted using the RNeasy Plus Micro kit (Qiagen). RNA was processed using the RNA-seq Poly A method, and 100 bp paired-end RNA-seq was performed on the Illumina HiSeq4000 platform (Wellcome Centre for Human Genetics, University of Oxford).

For data analysis, reads were aligned against the Ensembl transcriptome and genome (GRCh38.EBVB95-8wt.ERCC) using STAR (version 2.5.3a). The allocation of reads to protein-coding gene meta features was done using HTSeq. Differential expression analysis on genes, with at least one aligned fragment, was performed using general linear modeling in edgeR with trimmed-M-of-means library size correction and tagwise dispersion estimates. Differentially expressed genes were identified using the default edgeR threshold [false discovery rate (FDR) < 0.05]. Gene ontology analysis was performed using ClusterProfiler. The third biological replicate of the Δ*550* sample was removed as an outlier due to a low number of detected genes and initial exploratory analysis using multidimensional scaling of intersample differences.

### Single-cell RNA-seq of FACS-sorted TEC

Single-cell libraries were processed using Cellranger (version 3.1.0). Cells were excluded from analysis if the total unique molecular identifier (UMI) count was <1000 or >32,000; the number of detectable features was <562 or >7943, or the proportion of UMIs mapping to mitochondrial genes was >10%. Cells from individual 10X Chromium lanes were combined using canonical correlation analysis–based integration in Seurat (version 3.1.1) (*60*). Cell clusters were identified using the default resolution of 0.8 and visualized using Uniform Manifold Approximation and Projection (UMAP). Cell type annotation was based on projection from our previously published reference data (*1*), along with manual annotation of non-TEC clusters. Differential analysis was conducted in Seurat using FindMarkers with default settings. ClusterProfiler was used for gene ontology analysis (*61*).

### Electrophoretic mobility shift assay

Electrophoretic mobility shift assay (EMSA) was performed as recently described (*9*), using purified wild-type FOXN1 and Δ*550* FOXN1 constructs. The sequence of the DNA probe used was part of the minimal promoter region of the proteasome subunit alpha 7, a high-confidence FOXN1 target (*2*) with the following sequence: 5′GCAGCA**GACGC**AACAGAGCGA**GACGC**CAGGG3′ (with FOXN1 consensus sites in bold).

### ChIP followed by DNA sequencing

The 4D6 cells were transfected with a construct encoding either wild-type or the Δ550 FOXN1 mutant and tagged with a FLAG sequence. Three biological replicates of each of these two conditions were collected for ChIP-seq analyses. ChIP was performed using the iDeal ChIP-seq kit for transcription factors (Diagenode) as per the manufacturer’s recommendations. Briefly, 2.5 × 10^6^ transfected cells were collected and subjected to protein-DNA cross-linking for 20 min. Cells were then lysed, and chromatin was sheared for eight sonication cycles (30″ ON/30″ OFF). Sheared chromatin was immunoprecipitated using M2 anti-FLAG antibody (Sigma-Aldrich), while parallel input samples from nonimmunoprecipitated chromatin were prepared for each biological replicate of each condition. DNA libraries for all 12 samples were prepared using the MicroPlex Library Preparation Kit (12 indexes; Diagenode) following the manufacturer’s recommendations. Libraries were pooled and sequenced at the Wellcome Centre for Human Genetics, University of Oxford using 75-bp paired-end sequencing using an Illumina HiSeq4000 platform. For data analysis, contaminating adaptor sequences were removed from fastq sequences using Trimmomatic (version 0.32). Reads were aligned against the human genome (GRCh38.EBVB95-8wt.ERCC) using stampy (version 1.0.32). Peaks were called on deduplicated aligned sequences (paired ChIP and input samples) using MACS2 (version 2.0.10) with a relaxed *P* value setting of 0.1. Peaks from replicates were then pooled and analyzed using IDR analysis (IDR < 0.05) (https://sites.google.com/site/anshulkundaje/projects/idr). Peaks were filtered against the ENCODE blacklist regions (https://sites.google.com/site/anshulkundaje/projects/blacklists). De novo motif identification was performed using MEME-ChIP (*31*).

### MiSeq sequencing to determine the length of the CDR3 region of TCR α and β chains

RNA was extracted from PBMCs of the patient and an aged-matched control using the Qiagen RNA blood Mini kit (Qiagen). Deoxyribonuclease (DNase) treatment of the extracted RNA was performed (RQ1 DNAse Promega) following the manufacturer’s instructions to remove any residual genomic DNA. A library preparation was performed as previously described following a protocol for quantitative TCR sequencing (*62*). The prepared libraries were pooled at a final 12-pM concentration and sequenced using the Illumina MiSeq platform, using a version 2 chemistry 2x250 PE kit. Data were analyzed as previously described (*62*). Shannon entropy was used as a diversity measure of the data. As detailed in (*62*), Shannon entropy was calculated using the vegan package (version 2.4.2) to quantify diversity by incorporating both evenness and richness of a variable. The ggplot2 package (version 2.2.1) was used for all data visualizations.

### Immunoprecipitation of tagged FOXN1 variants

The 4D6 cells were transfected and cotransfected to express either FLAG-labeled wild-type, FLAG-labeled Δ550-FLAG, or FLAG and myc–labeled wild-type FOXN1 in the presence or absence of unlabeled Δ550 FOXN1. Cell lysates were incubated with anti-FLAG or anti-Myc antibodies in the presence of protein G or A magnetic beads (Thermo Fisher Scientific) for 2 hours at 4°C while slowly rotating. For pulldowns to be analyzed by Western blotting, beads were boiled at 95°C with denaturating sample buffer [250 mM tris-HCl (pH 6.8), 10% (w/v) SDS, 35% (v/v) glycerol, 0.05% bromophenol blue, and 0.7 M 2-mercaptoethanol]. For pulldowns to be analyzed by mass spectrometry, beads were incubated with disuccinimidyl sulfoxide (DSSO; Sigma-Aldrich) at a final concentration of 1 mM at room temperature for 45 min with shaking according to the manufacturer’s recommendations. The reaction was paused by adding 10 μl of tris-HCL (ph7.5) 1 M for 5 min, and beads were washed three times with PBS. Proteins were eluted from beads using 40 μg of 3× FLAG peptide (Merck) for 200 μl of immunoprecipitated sample incubated on a rotator for 45 min at 4°C.

### Live cell imaging

The 4D6 cells were plated in 35-mm glass bottom dishes (Ibidi) in the 4D6 cell growth medium described earlier and were then transfected with various combinations of the wild-type and mutant FOXN1 constructs. Twenty-four hours after transfection, the culture medium was exchanged with Leibovitz 15 medium lacking phenol red (Thermo Fisher Scientific), and cells were imaged with a ZeissLSM 880 inverted confocal laser scanning microscope. A laser wavelength of 488 nm was used to excite GFP or EYFP. Laser power was kept 50 μW or below to avoid phototoxicity. FOXN1 condensates were counted using the spot counter plug-in of the Fiji software. Default values for spot size and fluorescence were used for this analysis.

### Fluorescence recovery after photobleaching

FRAP was performed in defined areas of the nucleus of 4D6 cells transfected with wild-type, Δ550, Δ457, or Δ550b FOXN1 variants tagged with EYFP using a LSM 880 inverted confocal laser scanning microscope with 40× W (1.2) objective. Fluorescence recovery within the bleached area was normalized to the fluorescence within a reference area outside the bleached area. To calculate the recovery rate, normalized fluorescence recovery was further normalized to the mean prebleached intensity. Normalized data were fitted using the curve fitting function in Fiji. The following fitting formula was used: *F*(*t*) = *a*×(1 − exp(−*b*×*t*)) + *F*(0), where “*a*” is the fractional amplitude of a slowly recovering fraction, “*b*” is the recovery rate, and “*F*(0)” is the fractional fluorescence intensity at time zero. (*63*).

### FCS of wild-type and Δ*550* FOXN1

A LSM 780 inverted confocal laser scanning microscope with 40× W (1.2) objective was used. The microscope was calibrated before acquiring the data, and the point spread function was determined using 10 nM Alexa Fluor 488 solution. FCS on cells was performed in defined areas of the nucleus of 4D6 cells transfected with wild-type-FOXN1-GFP. A fluorescence image was first taken of the cell’s nuclei containing fluorescently labeled FOXN1, and subsequently, the nuclear location for the FCS analysis was selected, and changes in fluorescence were measured at this position as a function of time. For each selected position, five FCS measurements of 5 s each were taken. FCS curves from single runs were individually sorted to determine whether they met the designated criteria that stipulated that photobleaching during acquisition time is less than 10% (*64*). Curves that did not meet these criteria were excluded from further analysis. Selected FCS curves from each run were combined and fitted using the FoCuS-point software with a three-dimensional and triplet model (*65*).

### Mass spectrometry proteomics analysis

The 4D6 cells were transfected with constructs encoding either wild-type-FLAG or Δ550-FLAG sequences. Three experimental replicates were prepared for each experimental condition. Cells were lysed, and anti-FLAG immunoprecipitation was performed as described above. To confirm the presence of proteins other than FOXN1, a fraction of the sample was analyzed by silver staining (Pierce silver staining kit, Thermo Fisher Scientific) according to the manufacturer’s recommendations. For the mass spectrometric analysis, frozen samples were thawed and subjected to two rounds of chloroform/methanol precipitation. An in-solution trypsin digestion was performed as previously described (*66*). After digestion and desalting, samples were resuspended in 20 μl 2% acetonitrile and 0.1% formic acid and subjected to nano-flow LC-MS/MS (hereafter referred to as MS) (*66*). LC-MS/MS analysis was performed on an Orbitrap Fusion Lumos instrument (Thermo Fisher Scientific) coupled to an Ultimate 3000 nUPLC with an EASY-Spray column (50 cm). Peptides were separated using a solvent gradient of 2 to 35% acetonitrile in 0.1% formic acid/5% dimethyl sulfoxide for over 60 min. MS data were acquired using a method to also cater for DSSO cross-linked peptides (*67*). Briefly, precursor masses were acquired with a resolution of 60,000 for up to 50 ms. MS2 were acquired in the Orbitrap after collision induced dissociation (CID) fragmentation. A mass difference of 31.9721 triggered subsequent MS3 scans with increased collision energy (25 ➔ 35%) and detection in the linear ion trap in rapid mode followed by an MS2 scan after electron transfer dissociation (ETD) fragmentation and detection in the Orbitrap with a resolution of 15,000. The raw MS data were processed with Progenesis QI for Proteomics v. 4.1.6675.48614 to generate label-free relative quantitation. MS/MS spectra were searched against a SwissProt *Homo sapiens* database (retrieved 05/2018) with precursor mass tolerance of 10 ppm and fragment mass tolerance of 0.5 Da using Proteome Discoverer 3.5 including the XLinkX node (*68*). Variable modifications were set as oxidation on methionine and the DSSO water and tris quench monoadducts on lysine. Detected cross-links were not followed up. The raw quantitation values were further analyzed in R version 3.5.5. For each protein, correlation with FOXN1 was assessed by Spearman’s ⍴, and associated *P* values were calculated by the algorithm AS89 (*69*). FDR estimates at each *P* value cutoff (*q* values) were calculated from the list of *P* values using the fdrtool package (R package version 1.2.15. (https://CRAN.Rproject.org/package=fdrtool) (*70*). An FDR cutoff of 5% was applied to data analysis. The STRING protein-protein interaction database, i.e., a database of known and predicted protein-protein interactions containing information from experimental data, computational predictions, and public text collections https://string-db.org, was queried to construct a potential interaction network. The accession numbers of all proteins that were detected in all replicates of wild-type or Δ550 FOXN1 precipitates, that did not correspond to known contaminants (e.g., albumins, keratins, histones, cytoplasmic proteins, and ribosomal proteins) (*44*), and that localized to the nucleus according to the UniProt Knowledgebase (*45*) were submitted to the STRING database.

### EYFP^β5t^FOXN1^CMV^ 4D6 reporter cell line

#### Generation of the EYFP^β5t^FOXN1^CMV^ 4D6 reporter cell line

To generate the EYFP^β5t^FOXN1^CMV^ 4D6 reporter cell line, 4D6 cells were transduced with the in-house–designed lentivector EYFP^β5t^FOXN1^CMV^ that was commercially produced (Vector Builder). Stable integration of this lentivector into the genome of 4D6 cells allowed the constitutive expression of FOXN1 under the control of the CMV promoter.

The rationale for this construct was that functional FOXN1 bound to the β5t promoter (the same promoter sequence used for the luciferase assays described above) will control the expression of EYFP. Any changes in FOXN1-dependent transcriptional activity, for example, as a result of specific gene deletions or mutations in these cells, will hence be marked by changes in EYFP.

For the production of infectious lentivirus, 1.2 × 10^7^ human embryonic kidney–293 T cells were plated onto a 15-cm tissue culture dish. Twenty-four hours after plating, cells were cotransfected with the lentiviral transfer vector (EYFP^β5t^FOXN1^CMV^) and the psPAX2 and pMD2.G viral packaging vectors at a ratio of 4:3:2 using PEI-Pro (PolyPlus Transfection) following the manufacturer’s protocol. Culture medium was exchanged 6 hours after transfection with 10 ml of fresh Dulbecco’s modified Eagle’s medium (Thermo Fisher Scientific) complemented with 10% FBS, NEAA, penicillin-streptomycin, and l-glutamine. The lentiviral supernatant was collected at 48 and 72 hours after transfection, pooled and filtered with a 0.45-μm cellulose acetate syringe filter (Sartorius), and subsequently overlaid onto 5 ml of 20% sucrose and ultracentrifuged at 24,000 rpm for 2.5 hours (Beckman XPN80, SW32.Ti). The viral pellet was resuspended in PBS.

For the generation of the FOXN1-EYFP reporter cell line, 4D6 cells were virally transduced with the EYFP^β5t^FOXN1^CMV^ expression vector. A range of multiplicity of infection was used to determine the virus titer required to achieve a 5% transduction efficiency to ensure a single integration of the construct in successfully transduced cells. Transduction was performed in the presence of hexadimethrine bromide (8 μg/ml; Polybrene). The resulting stable cell line was evaluated 72 hours after transduction for EYFP expression levels using flow cytometry. Transduced cells were repetitively sorted by FACS to obtain a population with at least 75% EYFP-positive cells as successfully transduced cells displayed the tendency to decrease the frequency of EYFP-positive cells over time.

#### Cloning of sgRNAs in the Cas9 expression vector

Single-guide RNAs (sgRNAs) targeting FOXN1, YBX1, and CBP were selected from the GeneScript database https://genscript.com/gRNA-detail/1387/CREBBP-CRISPR-guide-RNA.html.

The following guide sequences were used: sgFOXN1, 5′CACCGTGCTCGTCATTTGTGTCCGA3′ (forward) and 5′AAAC TCGGACACAAATGACGAGCA C3′ (reverse); sg CBP, 5′CACCGGAATCACATGACGCATTGTC3′ (forward) and 5′AAACGACAATGCGTCATGTGATTCC3′ (reverse); sg YBX1, 5′CACCGGGACCATACCTGCGGAATCG3′ (forward) and 5′AAACCGATTCCGCAGGTATGGTCCC3′ (reverse). Each pair of guides was cloned into the Cas9-2A-mRuby2 vector (https://addgene.org/110164/ Addgene no. 110164) following the protocol of the Feng Zhang laboratory (https://media.addgene.org/cms/filer_public /e6/5a/e65a9ef8-c8ac-4f88-98da-3b7d7960394c/zhang-lab-general-cloning-protocol.pdf). A single colony of transformed bacteria per guide was selected, picked, grown overnight in liquid cultures, miniprepped, and eventually sent for sequencing to confirm the successful ligation into the Cas9-2A-mRuby2 vector.

#### CRISPR-mediated deletion of selected genes in the EYFP^β5t^FOXN1^CMV^ 4D6 reporter cell line

The CRISPR-mediated deletion of selected genes was performed by transfecting 4D6 EYFP^β5t^FOXN1^CMV^ cells with the respective guides using Fugene. Three technical replicates were prepared per sample. Cells transfected only with the Cas9-2A-mRuby2 vector or the sgCCR5 (a guide targeting a gene irrelevant for FOXN1 function) served as negative controls. Forty-eight hours after transfection, cells were collected in FACS buffer [1× PBS and 5% fetal bovine serum (FBS)], stained with DAPI for live/dead cell discrimination, and analyzed by flow cytometry. Live cells, DAPI negative, were gated for mRuby2 positivity (detected at 561 of 564 nm) to select successfully transduced cells. Last, the frequency of EYFP positivity was determined for cells that were mRuby2 positive. A decrease in EYFP positivity in cells transfected with the guide RNAs was used as a means to demonstrate the impact of the CRISPR-mediated deletion in overall FOXN1 functionality. The changes in EYFP positivity after transfection with the CRISPR guides for *Cbp*, *Ybx1*, *Foxn1*, and, as a control, *Ccr5* were calculated relative to the EYFP levels detected in cells transfected with only the Cas9-2A-mRuby2 vector.

The efficiency of CRISPR-mediated deletion of *Cbp*, *Ybx1*, and *Foxn1* in EYFP^β5t^FOXN1^CMV^ cells was assessed by qRT-PCR. Briefly, 4D6 EYFP^β5t^FOXN1^CMV^ cells were transfected with a Cas9-2A-mRuby vector alone or together with specific guides to target *Cbp*, *Ybx1*, and *Foxn1*. Forty-eight hours after transfection, cells were collected in FACS buffer (1× PBS and 5% FBS), stained with DAPI for live/dead cell discrimination, and analyzed by flow cytometry. Live, mRuby2-positive cells were flow cytometrically sorted, and RNA was extracted. qRT-PCR analysis was used to compare transcripts for CBP, YBX1, and FOXN1 among the differently transfected cells, and GAPDH transcripts were used as an internal reference for normalization using the *2^-ΔCT^* method.

### Mice

Animals were maintained under specific pathogen–free conditions, and experiments were performed according to institutional and U.K. Home Office regulations. Mouse colonies were maintained at the University of Oxford Biomedical Science facilities. Age- and gender-matched wild-type mice were used in all experiments as a reference for genetically modified animals.

Mice heterozygous for a *Foxn1* allele with a single nucleotide loss at position 1470 (designated FOXN1^WT/Δ505^) were generated at the Genome Engineering Facility of the MRC Weatherall Institute of Molecular Medicine, University of Oxford, using the CRISPR-Cas9–assisted mouse embryonic stem cell (mESC) targeting. In detail, murine JM8.1 C57B/6J ES cells were transfected with the pX459 plasmid construct that allowed expression of a Puromycin resistance gene, the Cas9 endonuclease, and sgRNA targeting the region in which the base pair change was expected to take place (Addgene 62988). The single-stranded donor oligonucleotide (ssODN) for homology-directed repair was also cotransfected to allow homologous integration of the desired genetic change. The ssODN was complementary to exon 8 of the mouse *Foxn1* locus with a single base pair deletion of an adenine at cDNA position 1370, and it also contained a de novo restriction enzyme site (Pvu II) that was inserted in the sequence without changing the codon usage. Successfully targeted mESC clones, verified by cloning and Sanger sequencing, were injected into the blastocysts of albino C57BL/6BrdCrHsd-Tyrc mice. Resultant chimeric animals were selected by the presence of black coat color and confirmed by genotyping. Male chimeras were mated with albino C57BL/6BrdCrHsd-Tyrc female mice to establish germline transmission. Successful integration of the mutant allele was ultimately verified by genotyping. Heterozygous Δ*505* mice were mated with C57BL/6J WT black mice to expand the colony. For genotyping the FOXN1^WT/Δ505^ mice, genomic DNA was extracted from ear clips and amplified by PCR using the relevant forward (FP: 5′AAACTGGGCTCTCCGCTGCTG3′) and reverse (RP: 5′AGTAGAGTATCGTGCATGGTCCTGG3′) primers [Taq polymerase (Sigma-Aldrich)] at an annealing temperature (*T*_m_) 64°C. PCR amplicons were digested using Pvu II (New England Biolabs) restriction enzyme at 37°C for 1 hour and were run on an agarose gel. The undigested PCR amplicon (uncut) was detected at 389-bp size. Pvu II digested amplicons from FOXN1^WT/WT^ mice generated two bands of 389- and 70-bp size, respectively, whereas Pvu II digested amplicons from FOXN1^WT/Δ505^ heterozygous mice generated two additional bands of 213- and 97-bp sizes, respectively.

For FTP^FOXN1^ mice, to probe Foxn1 promoter activity, C57BL/6 mice were rendered transgenic for the expression of an FTP with a slow conversion kinetic (*46*) under the transcriptional control of the endogenous Foxn1 locus. Briefly, the mCherry variant was inserted upstream of endogenous ATG start codon in exon 2. To engineer the targeting vector, homology arms were generated by PCR using bacterial artificial chromosome clones RP24-306F3 and RP23-204B15 from the C57BL/6J library as templates. In the targeting vector, the Neo cassette was flanked by self-deletion anchor sites. Diphtheria toxin A was used for negative selection of mESC clones successfully targeted.

### TEC and thymocyte isolation

Adipose and other tissues were removed from isolated thymi, which were subsequently subjected to three rounds of enzymatic digestion for which cut-up lobes were incubated at 37°C in Liberase digestion buffer [PBS Gibco 1X, Liberase Roche (2.5 mg/ml), and DNase Roche (10 mg/ml)]. Cells were incubated with anti-CD45 beads (Miltenyi Biotec) as per the manufacturer’s recommendations and subjected to CD45 depletion using the “depleteS” program on the AutoMACS separator (Miltenyi Biotec). CD45 depleted fractions were stained in PBS supplemented with 2% FBS (both Sigma-Aldrich) for flow cytometric analysis.

For thymocyte analysis, cells were obtained by gentle disruption of thymic lobes using frosted glass slides (Thermo Fisher Scientific). Cells were filtered through a 70-μm filter (Greiner) and washed with ice-cold PBS supplemented with 2% FBS before being stained for downstream flow cytometric analysis using the reagents listed in table S4. Extracellular antibody stains were conducted for 20 min at 4°C in the dark. CCR7 staining was performed at 37°C water bath for 30 min. The *Ulex europaeus* agglutinin I (UEA I) lectin (Vector Laboratories) was used pure, followed by anti-UEA1 biotin staining with secondary streptavidin-BV605. For the identification of dead cells, the Live/Dead Fixable Aqua stain kit (Thermo Fisher Scientific) was used. For flow cytometric phenotyping and cell sorting, a BD FACS Aria III (BD Biosciences) was used.

### Statistical analysis

Statistical analyses were performed as indicated in the figure legend using GraphPad Prism. For all analyses, *P* < 0.05 was statistically significant and labeled by *P < 0.05, **P <0.01, ***P 0.001, and ****P < 0.0001. No statistically significant differences were indicated by the abbreviation ns. Details on the number of biological or technical replicates are indicated in each figure legend. Data are summarized as bar graphs with mean value and SD indicated. Individual data points shown as dots in bar graphs represent biological replicates in all cases with the notable exceptions of the luciferase assays in Figs. 1 (C, E, and F) and 4A and fig. S9A (relative quantification of the protein bands), where dots represent technical replicates. Statistical analysis of sequencing data was performed as described in the relevant section in the Materials and Methods and in each figure legend.

## Notes

View/request a protocol for this paper from *Bio-protocol*.
