# Supplementary material for: FOXN1 forms higher-order nuclear condensates displaced by mutations causing immunodeficiency
Source: Sci Adv. 2021 Dec 3;7(49):eabj9247. doi: 10.1126/sciadv.abj9247 (PMC8641933; doi:10.1126/sciadv.abj9247)
Supplement: Supplementary file 1 — Figs. S1 to S9 Tables S3 and S4 [file sciadv.abj9247_sm.pdf]

Science Advances
AAAS

Supplementary Materials for

FOXN1 forms higher-order nuclear condensates displaced by mutations causing immunodeficiency

Ioanna A. Rota, Adam E. Handel, Stefano Maio, Fabian Klein, Fatima Dhalla, Mary E. Deadman, Stanley Cheuk, Joseph A. Newman, Yale S. Michaels, Saulius Zuklys, Nicolas Prevot, Philip Hublitz, Philip D. Charles, Athina Soragia Gkazi, Eleni Adamopoulou, Waseem Qasim, Edward Graham Davies, Imelda Hanson, Alistair T. Pagnamenta, Carme Camps, Helene M. Dreau, Andrea White, Kieran James, Roman Fischer, Opher Gileadi, Jenny C. Taylor, Tudor Fulga, B. Christoffer Lagerholm, Graham Anderson, Erdinc Sezgin, Georg A. Holländer*

*Corresponding author. Email: georg.hollander@paediatrics.ox.ac.uk

Published 3 December 2021, *Sci. Adv.* **7**, eabj9247 (2021)
DOI: 10.1126/sciadv.abj9247

The PDF file includes:

Figs. S1 to S9
Tables S3 and S4

Other Supplementary Material for this manuscript includes the following:

Tables S1 and S2

Figure S1

A)

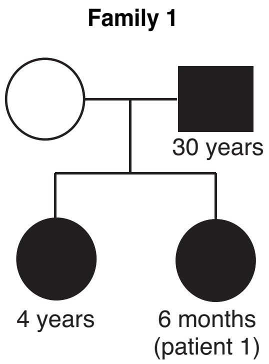

B)

	α chain		β chain	
	patient1	control	patient1	control
number of unique CDR3 regions	7777	13877	10229	18266
TCR diversity	10.237	12.716	11.001	13.228

C)

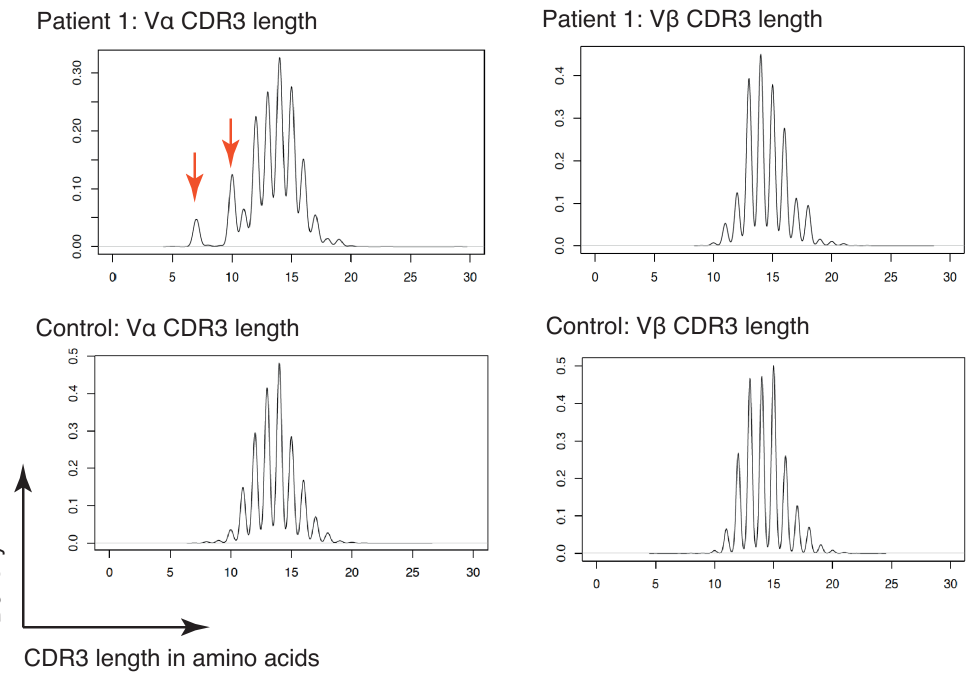

D)

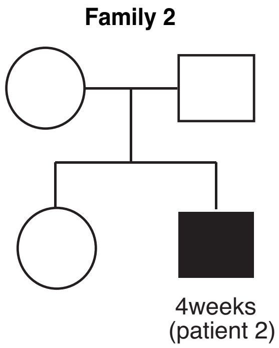

E)

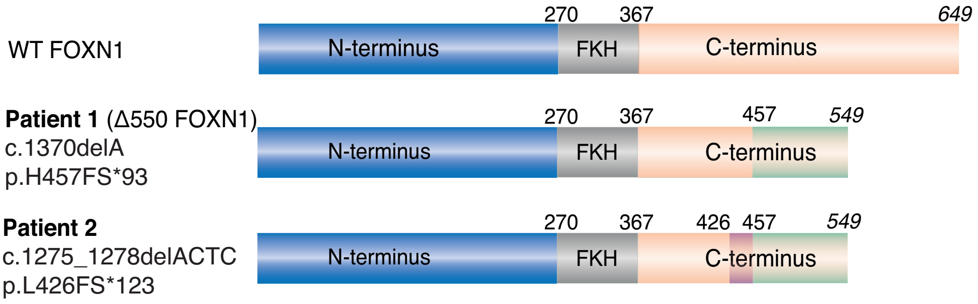

F)

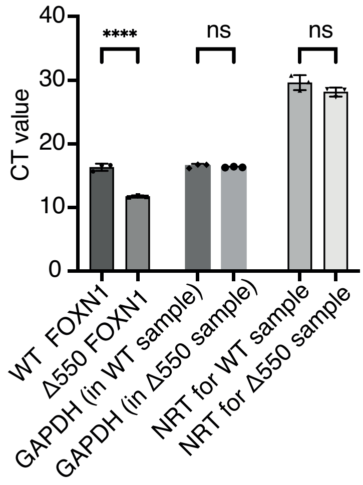

Figure S1. Clinical and experimental data of patients with novel FOXN1 variants
(A) Family pedigree of the index patient with the Δ550 FOXN1 mutation. Other chromosomal rearrangements or abnormalities in the patients were excluded by Comparative Genomic Hybridization array (CGH). Moreover, other known genetic variants associated with primary immunodeficiencies (PID) were not found in any of the individuals. The index patient was initial brought to medical attention due to severe dermatitis, a history of chronic diarrhea and an acute chest infection caused by rhinovirus. Peripheral blood testing showed a T cell lymphopenia (32% of total lymphocytes; normal range 51–77%.) with absent T cell receptor excision circles (TRECs). A chest ultrasound revealed the absence of a thymus. Immunological diagnostics demonstrated normal T cell proliferative responses to phytohemagglutinin as well as normal total serum IgG levels. The 4-year-old sister also had a reduction in naïve T cells, absent TRECs and a missing thymus but was otherwise thriving and clinically well. The 30-year-old father also lacked TRECs, but he was clinically well. Both the father and the sister also had high Epstein-Barr virus (EBV) serum loads but neither had developed any further symptoms. Following the initial clinical presentation, the index patient remained well and did not require further medical attention. It is of note that neither alopecia nor nail dystrophy were noted in the index patient, the older sister or their father. (B) Reduced alpha and beta chain TCR diversities compared to an age-matched control. The table shows the total number of unique CDR3 regions and TCR diversity as calculated by the Shannon entropy. Low Shannon indices reveal lower diversity (63) (C) CDR3 length in amino acids of TCR alpha and beta chains of peripheral T cells isolated from patient 1 and an age-matched control as assessed by MiSeq. The red arrows indicate oligoclonal expansion of T cells with CDR3 length of 7 and 10 amino acids respectively. (D) Family pedigree of a second patient with a FOXN1 variant leading to a scrambled sequence largely identical to the Δ550 FOXN1 mutation and a premature translational stop identical to that of the Δ550

FOXN1 variant. Similar to Patient 1, patient 2 was heterozygous for this FOXN1 mutation and clinically presented with athymia, T cell lymphopenia and absent T cell receptor excision circles (TRECs). Neither alopecia nor nail dystrophy were noted in Patient 2. Squares in panel (A) and (D) represent male and circles female family members. Filled symbols indicate individuals with the heterozygous FOXN1 mutation. (E) Schematic representation of wild-type (wt), Δ550 FOXN1 and FOXN1 variant found in patient 2. FKH: forkhead domain, numbers represent amino acids. The purple coloured box represents the amino acid sequence unique to patient 2 and the green box indicated sequences identical in patient 1 and 2. Numbers in italics indicate the position of the stop codon. (F) Ct values of FOXN1 and GAPDH from qRT-PCR analysis of 4D6 cells transfected with WT or Δ550 FOXN1. The Ct values of a No-Reverse-Transcriptase (NRT) control for both the WT and the Δ550 sample are also shown. GAPDH was not detected in the NRT controls proving the successful elimination of genomic DNA. Mean and SD is indicated, unpaired t-test 0.05 (ns), <0.0001(****),

Figure S2

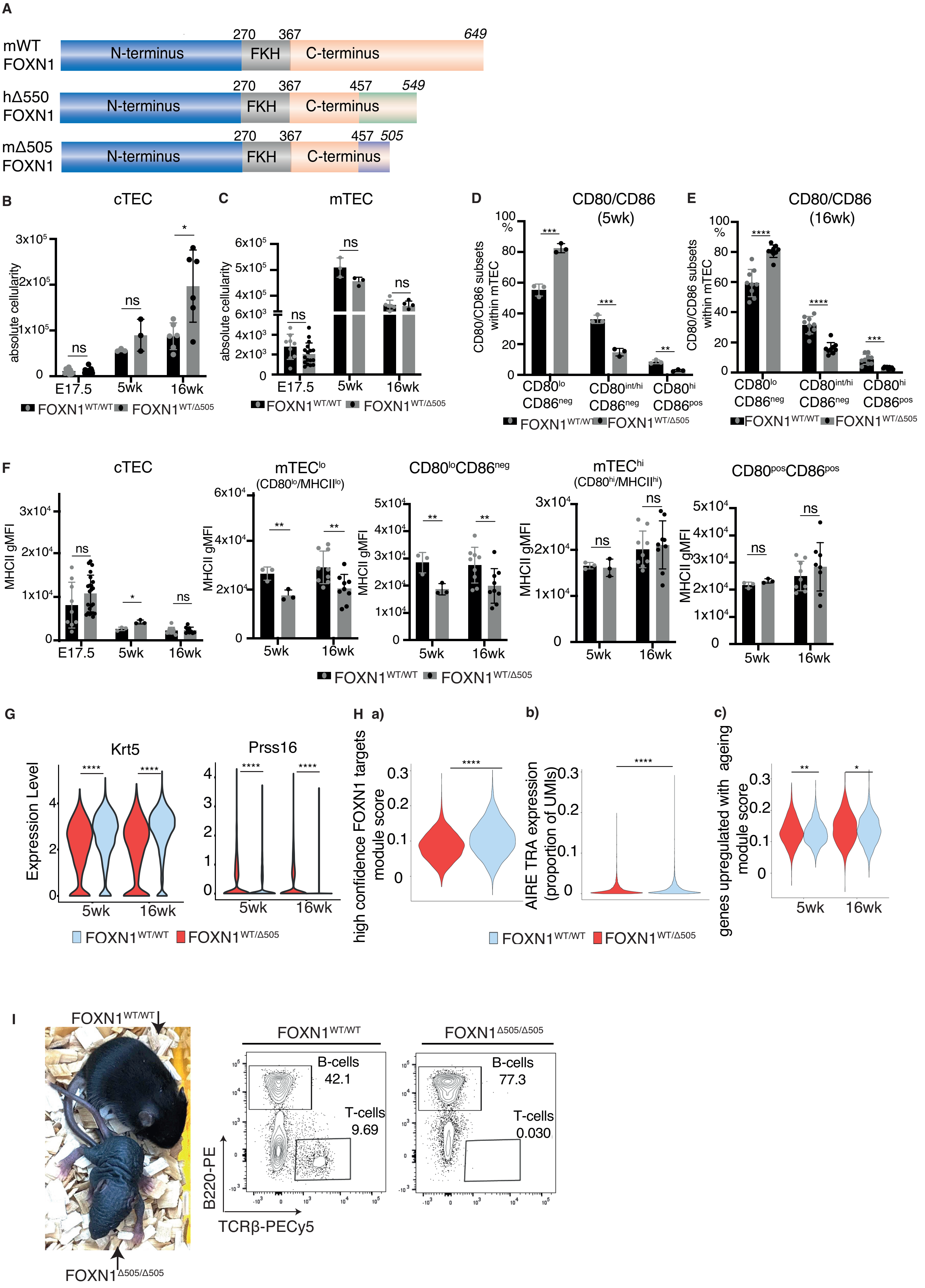
A
mWT
FOXN1
N-terminus
FKH
C-terminus
270
367
649
hΔ550
FOXN1
457
549
mΔ505
FOXN1
505
B
cTEC
absolute cellularity
3x10^5
2x10^5
1x10^5
0
ns
*
E17.5
5wk
16wk
FOXN1 WT/WT
FOXN1 WT/Δ505
C
mTEC
6x10^5
5x10^5
4x10^5
6x10^3
4x10^3
2x10^3
D
CD80/CD86
(5wk)
CD80/CD86 subsets
within mTEC
100
%
80
60
40
20
***
**
CD80lo
CD86neg
CD80int/hi
CD86neg
CD80hi
CD86pos
E
CD80/CD86
(16wk)
****
F
cTEC
MHCII gMFI
3x10^4
2x10^4
1x10^4
mTEClo
(CD80lo/MHCIIlo)
6x10^4
4x10^4
CD80loCD86neg
5x10^4
mTEChi
(CD80hi/MHCIIhi)
CD80posCD86pos
G
Krt5
Prss16
Expression Level
H a)
high confidence FOXN1 targets
module score
0.3
0.2
0.1
b)
AIRE TRA expression
(proportion of UMIs)
c)
genes upregulated with ageing
module score
I
FOXN1 WT/WT
FOXN1 Δ505/Δ505
B-cells
42.1
T-cells
9.69
B-cells
77.3
T-cells
0.030
B220-PE
TCRβ-PECy5

Figure S2: Mice heterozygous for the Δ505 mutation exhibit a partial block in TEC differentiation and differences in gene enrichment

(A)Schematic representation of mouse wild-type (mWT), human Δ550 (Δ550) and mouse Δ505 (mΔ505) FOXN1. FKH: forkhead domain. Green and blue boxes represent the scrambled in the human and mouse FOXN1 sequence, respectively. Numbers in italics indicate the position of the stop codon. (B-E) Analysis of FOXN1$^{WT/WT}$ and FOXN1$^{WT/\Delta505}$ mice at the indicated ages for cTEC, mTEC, and CD80^{+}CD86^{+} mTEC cellularity. (F) Level of MHC cell surface expression on cTEC, MHC^{low} mTEC, CD80^{low}CD86^{-} mTEC, MHC^{high} mTEC and CD80^{+}CD86^{+} mTEC of FOXN1$^{WT/WT}$ and FOXN1$^{WT/\Delta505}$ mice at the indicated ages. (G) Violin plots with expression levels of *Krt5*, *Prss16* at 5 and 16-week-old intertypical TEC. (H) Violin plots of data from mature mTEC: (a) Expression of FOXN1 high confidence target genes, (b) the proportion of unique molecular identifiers (UMIs) assigned to AIRE-induced genes and (c) the expression of genes upregulated in a tissue-independent manner throughout ageing (I) Phenotype of FOXN1$^{\Delta505/\Delta505}$ mice. Photograph of a hairless FOXN1$^{\Delta505/\Delta505}$ mouse next to a FOXN1$^{WT/WT}$ littermate. FACs plots showing the absence of peripheral T cells in FOXN1$^{\Delta505/\Delta505}$ mice. Data is from 4 (E17.5), 1 (week 5) and 2 (week 16) independent experiments with at least 3 FOXN1$^{WT/WT}$ and 3 FOXN1$^{WT/\Delta505}$ male mice for (B- F). (G) data is from 3 FOXN1$^{WT/WT}$ and 3 FOXN1$^{WT/}$Δ505 mice at 5 and 16 weeks of age, 22,228 interypical TEC analysed (H) The data is from 3 FOXN1$^{WT/WT}$ and 3 FOXN1$^{WT/\Delta505}$ mice: 6,687 mature mTEC, (I) data is from an independent experiment comparing FOXN1$^{WT/WT}$ and FOXN1$^{\Delta505/\Delta505}$ mice. Mean and SD is indicated ≥0.05 (ns), *<0.05, ** <0.01, ***< 0.001 and ****<0.0001, two-tailed unpaired t-test for (B-F), Wilcoxon test for (G-H).

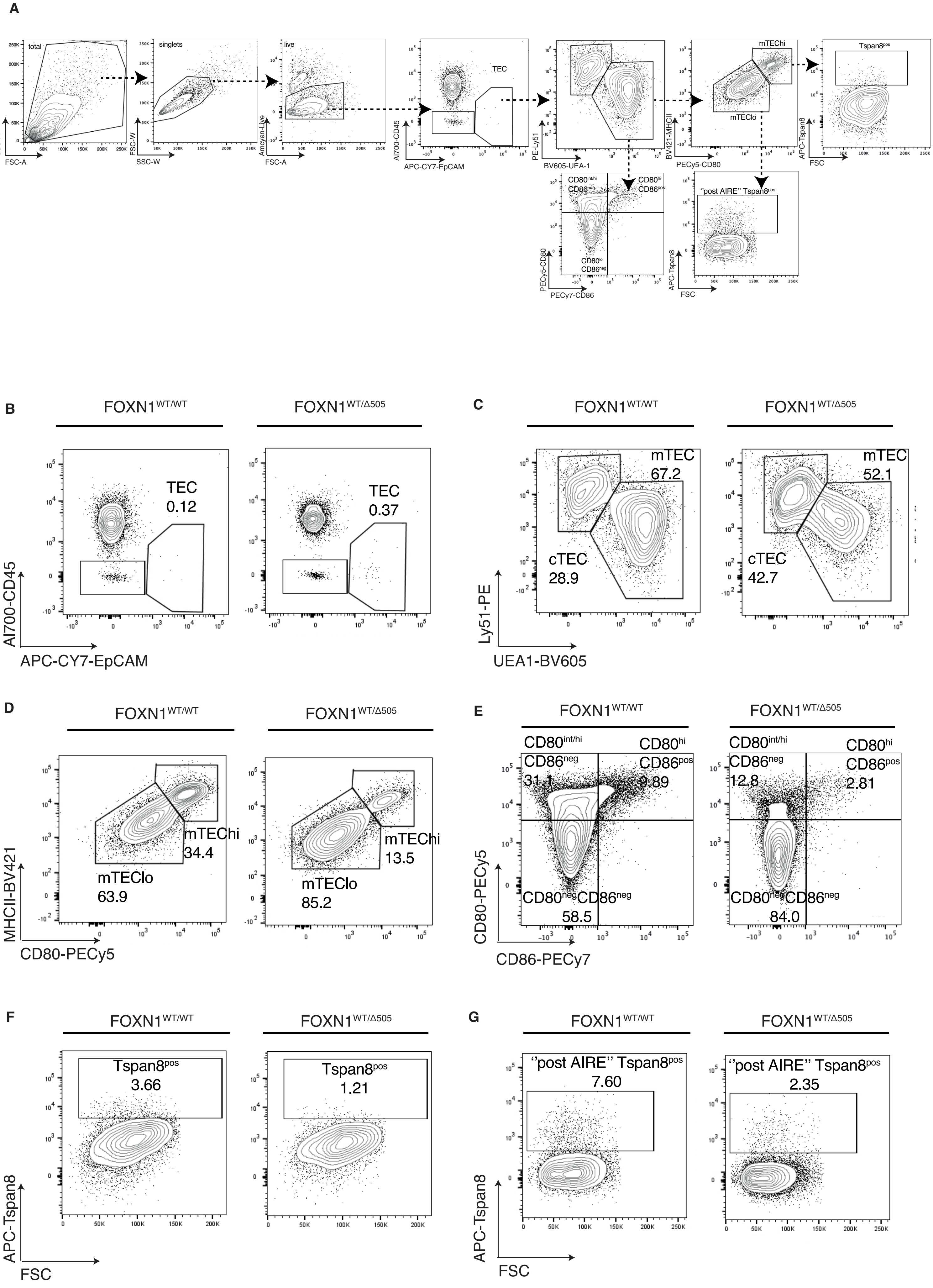
A
total
singlets
live
TEC
mTEChi
mTEClo
Tspan8pos
"post AIRE" Tspan8pos
SSC-A
FSC-A
FSC-W
SSC-W
Amcyan-Live
Al700-CD45
APC-CY7-EpCAM
PE-Ly51
BV605-UEA-1
BV421-MHCII
PECy5-CD80
APC-Tspan8
FSC
PECy7-CD86
B
FOXN1WT/WT
FOXN1WT/Δ505
TEC
0.12
TEC
0.37
Al700-CD45
APC-CY7-EpCAM
C
FOXN1WT/WT
FOXN1WT/Δ505
mTEC
67.2
cTEC
28.9
mTEC
52.1
cTEC
42.7
Ly51-PE
UEA1-BV605
D
FOXN1WT/WT
FOXN1WT/Δ505
mTEChi
34.4
mTEClo
63.9
mTEChi
13.5
mTEClo
85.2
MHCII-BV421
CD80-PECy5
E
FOXN1WT/WT
FOXN1WT/Δ505
CD80int/hi CD86neg 31.1
CD80hi CD86pos 9.89
CD80neg CD86neg 58.5
CD80int/hi CD86neg 12.8
CD80hi CD86pos 2.81
CD80neg CD86neg 84.0
CD80-PECy5
CD86-PECy7
F
FOXN1WT/WT
FOXN1WT/Δ505
Tspan8pos
3.66
Tspan8pos
1.21
APC-Tspan8
FSC
G
FOXN1WT/WT
FOXN1WT/Δ505
"post AIRE" Tspan8pos
7.60
"post AIRE" Tspan8pos
2.35
APC-Tspan8
FSC

Figue S3: Gating strategy and representative FACs plots of TEC subsets in FOXN1$^{WT/WT}$ and FOXN1$^{\Delta 505/WT}$ mice

(A) gating strategy for the identification of TEC subsets. FACs plots from 16 week old FOXN1$^{WT/WT}$and FOXN1$^{\Delta 505/WT}$mice comparing (B) total TEC (C)mTEC and cTEC (D)mTEChi and mTEClo (E) CD80,CD86 subpopulations (F) Tspan8^{pos} within mTEC^{hi} (G) Tspan8^{pos}within mTEC^{lo}

Figure S4

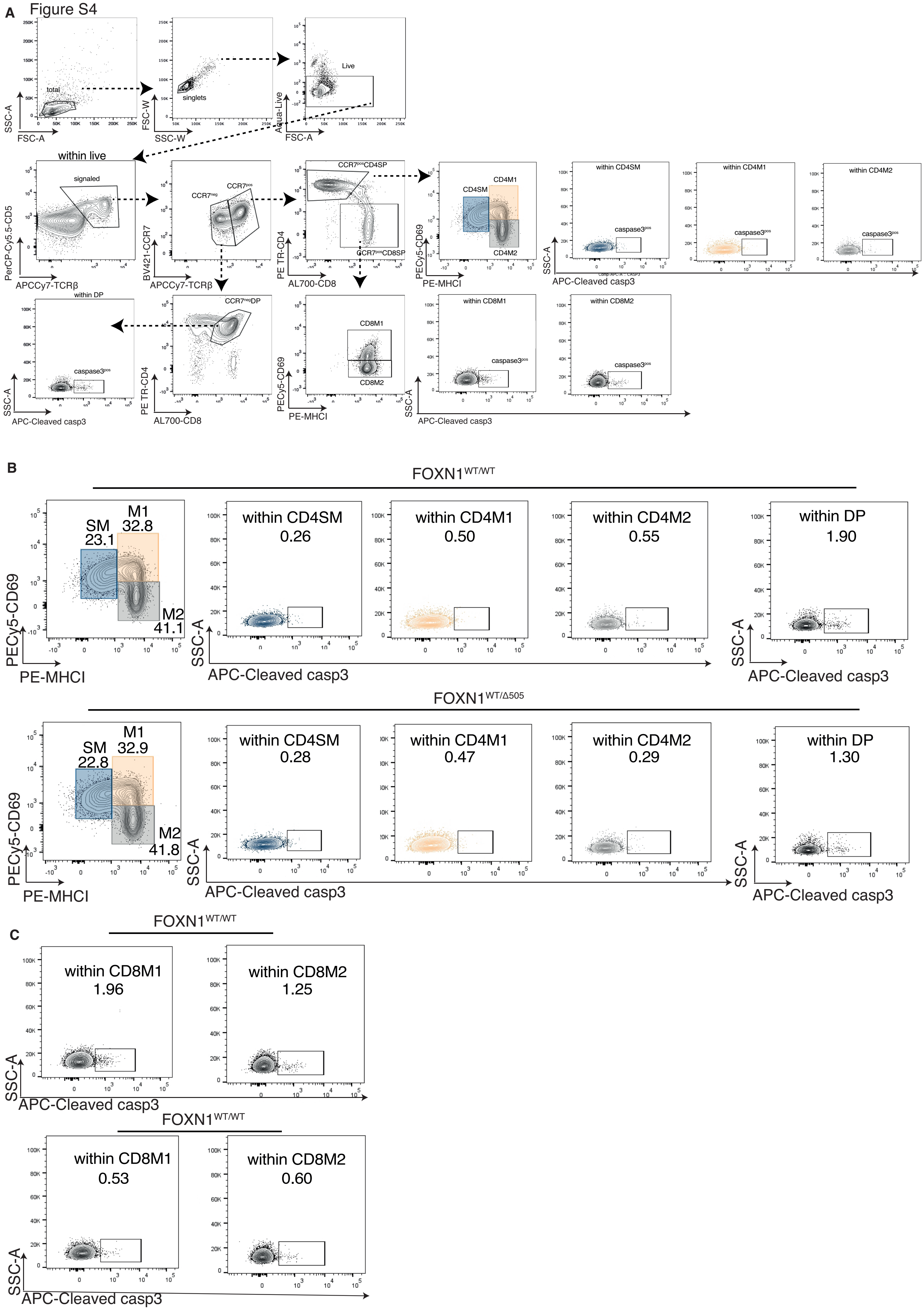

A
total
singlets
Live
SSC-A
FSC-A
FSC-W
SSC-W
Aqua-Live
within live
signaled
PerCP-Cy5.5-CD5
APCCy7-TCRβ
CCR7neg
CCR7pos
BV421-CCR7
CCR7posCD4SP
CCR7posCD8SP
PE TR-CD4
AL700-CD8
CD4M1
CD4SM
CD4M2
PECy5-CD69
PE-MHCI
within CD4SM
within CD4M1
within CD4M2
caspase3pos
APC-Cleaved casp3
within DP
CCR7negDP
CD8M1
CD8M2
within CD8M1
within CD8M2
B
FOXN1WT/WT
SM
23.1
M1
32.8
M2
41.1
within CD4SM
0.26
within CD4M1
0.50
within CD4M2
0.55
within DP
1.90
FOXN1WT/Δ505
SM
22.8
M1
32.9
M2
41.8
within CD4SM
0.28
within CD4M1
0.47
within CD4M2
0.29
within DP
1.30
C
FOXN1WT/WT
within CD8M1
1.96
within CD8M2
1.25
FOXN1WT/WT
within CD8M1
0.53
within CD8M2
0.60

Figure S4: Gating strategy and representative FACs plots assessing thymocyte clonal deletion in FOXN1$^{WT/WT}$and FOXN1$^{\Delta 505/WT}$mice using the cleaved caspase 3 marker

(A) gating strategy for the assessment of thymocyte clonal deletion. FACs plots from 16 week old FOXN1$^{WT/WT}$and FOXN1$^{\Delta 505/WT}$mice comparing (B) the frequency of cleaved caspase 3 positive cells within DP and SPCD4SM, SPCD4M1 and SPCD4 M2 thymocyte subsets (C) the frequency of cleaved caspase 3 positive cells within SPCD8 M1 and SPCD8M2 thymocyte subsets

Figure S5

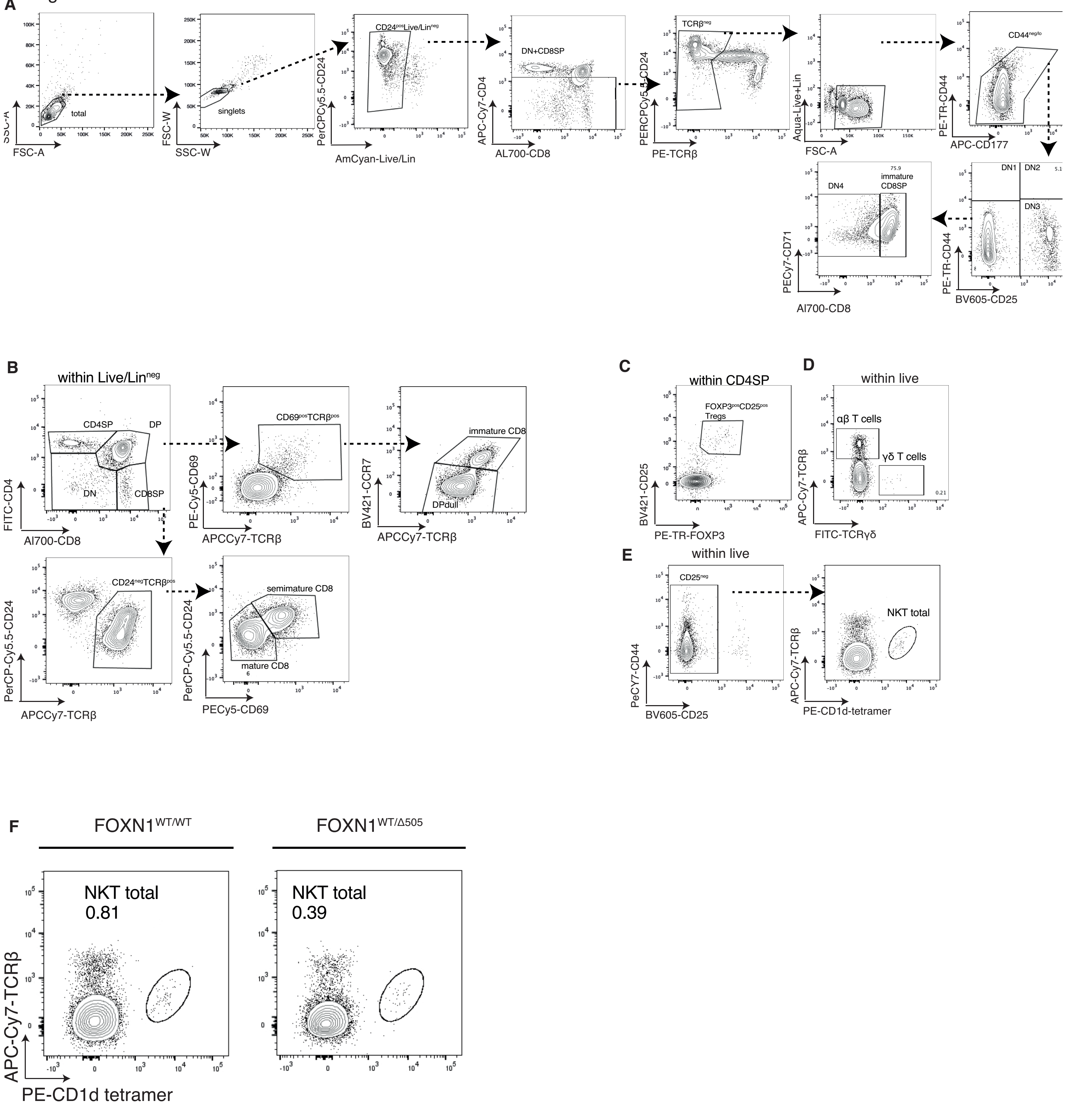

Figure S5: Gating strategy and representative FACs plots of thymocyte subsets in FOXN1$^{WT/WT}$and FOXN1$^{\Delta 505/WT}$mice.

Gating strategy for the identification of (A) DN thymocyte subpopulations (B) SPCD8 maturation stages (C) FOXP3^{pos} CD25^{pos} Tregs (D)γδ Tcells (E)NKT cells (F) FACS plots from 16 weeks old FOXN1$^{WT/WT}$and FOXN1$^{\Delta 505/WT}$mice comparing the NKT cell subset

Figure S6

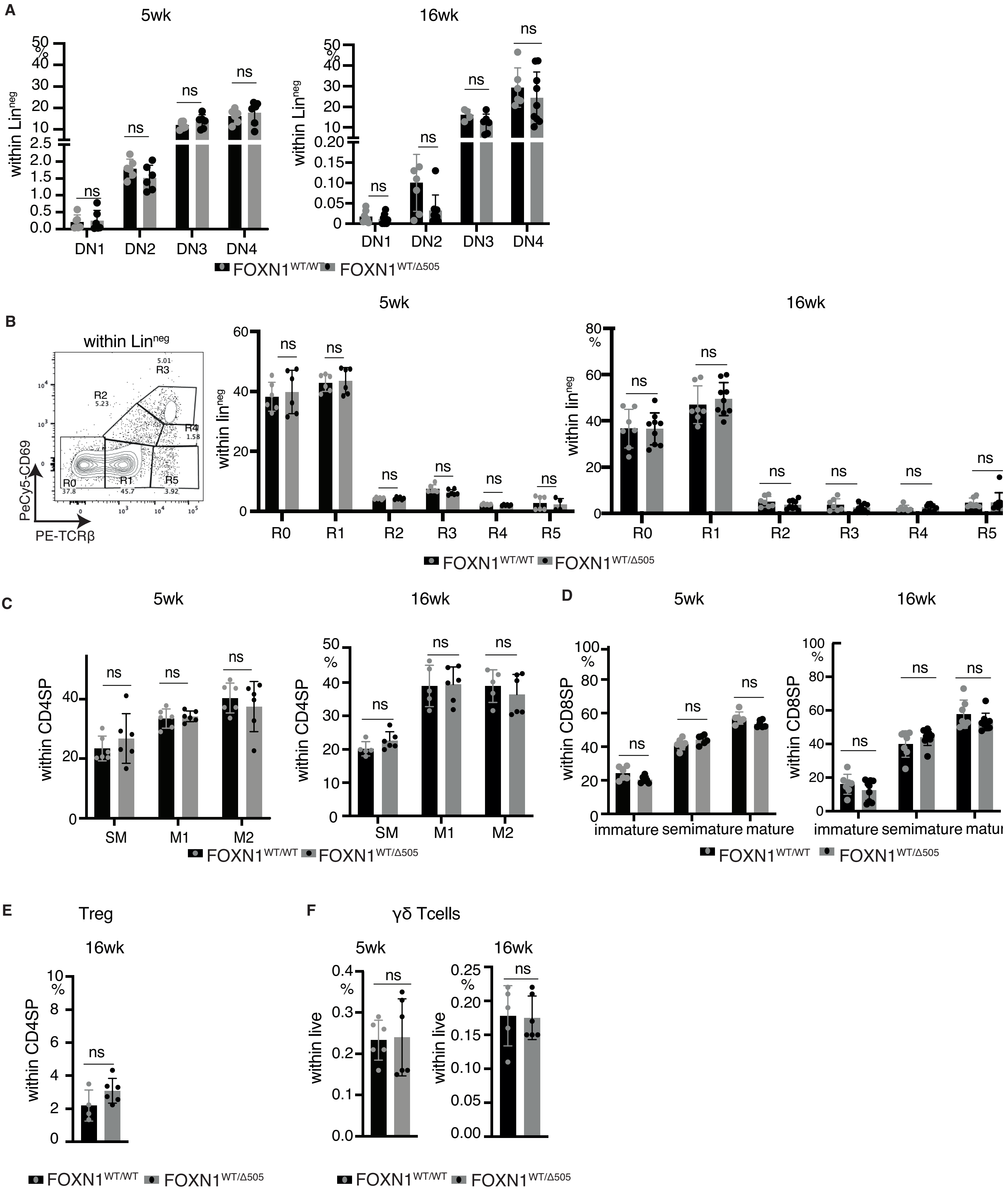
A
5wk
16wk
within Lin^neg %
ns
DN1
DN2
DN3
DN4
FOXN1 WT/WT
FOXN1 WT/Δ505
B
within Lin^neg
R0
R1
R2
R3
R4
R5
PeCy5-CD69
PE-TCRβ
5wk
16wk
within lin^neg %
ns
C
5wk
16wk
within CD4SP %
SM
M1
M2
D
within CD8SP %
immature
semimature
mature
E
Treg
16wk
within CD4SP %
F
γδ Tcells
5wk
16wk
within live %
FOXN1 WT/WT
FOXN1 WT/Δ505

Figure S6: Thymocyte development in mice heterozygous for Δ505 FOXN1.

Thymocyte analysis in 5 and 16 week old FOXN1$^{WT/WT}$ and FOXN1$^{WT/\Delta 505}$ mice. (A) Lineage-negative (Lin-) CD4^{-}CD8^{-} (double negative, DN) thymocytes were stained for their cell surface expression of CD44 and CD25 defining 4 separate subpopulations: DN1 (CD44^{+}CD25^{-}), DN2 (CD44^{+}CD25^{+}), DN3 (CD44^{-}CD25^{-}) and DN4 (CD44^{-}CD25^{-}). Lin- was defined as negative for the expression of CD11b, CD11c, Gr1, CD19, CD49b, F4/80, NK1.1, TCRγδ, and Ter119. (B) Thymocyte maturational stages based on TCR and CD69 cell surface expression. FACs plot shows analysis of wild-type thymocytes indicating the chosen gates R0-R5. Bar graphs show frequency of indicated thymocyte subpopulations at 5 and 16 weeks of age. (C) Stages of CD4SP thymocytes. The CD4SP cells were grouped into semi-mature (SM: CD69^{+}MHC I^{low}), mature 1 (M1: CD69^{+}MHC I^{+}) and mature 2 (M2: CD69^{-} MHC I^{+}) thymocytes [64]. (D) Stages of CD8SP thymocytes. The CD8SP cells were grouped into immature (CCR7^{pos}, TCRβ^{pos}, CD69^{pos} within DP), semimature (CD24^{pos}, CD69^{pos} within CD8SP) and mature (CD24^{neg}, CD69^{neg} within CD8SP) thymocytes. (E) Frequency of regulatory T cells (Treg: FOXP3^{+}CD25^{+} CD4SP) (F) Frequency of γδ T cells. Each symbol represents data from an individual wild-type or mutant mouse at the indicated age. Data is from 3 (panels A,D) and 2 independent experiments (panels C, E, and F). Mean value and SD are shown and were calculated by two-tailed unpaired t-test. p-values: ≥ 0.05 (ns). The flow cytometric gating strategies are shown in Figure S4 and S5.

Figure S7

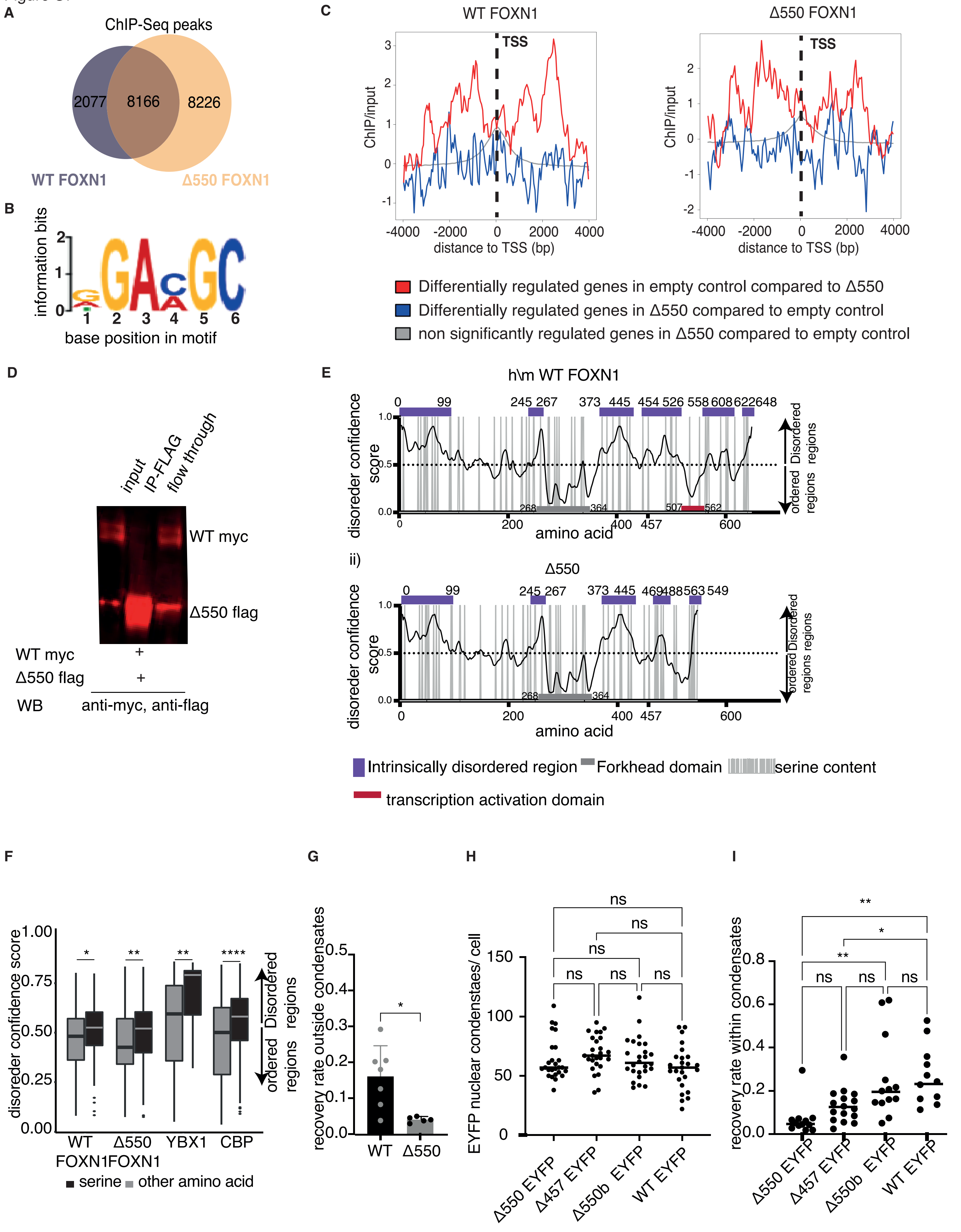

A
ChIP-Seq peaks
2077
8166
8226
WT FOXN1
Δ550 FOXN1
B
information bits
base position in motif
C
WT FOXN1
Δ550 FOXN1
TSS
ChIP/input
distance to TSS (bp)
Differentially regulated genes in empty control compared to Δ550
Differentially regulated genes in Δ550 compared to empty control
non significantly regulated genes in Δ550 compared to empty control
D
input
IP-FLAG
flow through
WT myc
Δ550 flag
WT myc +
Δ550 flag +
WB anti-myc, anti-flag
E
h\m WT FOXN1
disoreder confidence score
amino acid
Disordered regions
ordered regions
ii)
Δ550
Intrinsically disordered region
Forkhead domain
serine content
transcription activation domain
F
disoreder confidence score
WT FOXN1
Δ550 FOXN1
YBX1
CBP
serine
other amino acid
G
recovery rate outside condensates
WT
Δ550
H
EYFP nuclear condensates/ cell
ns
Δ550 EYFP
Δ457 EYFP
Δ550b EYFP
WT EYFP
I
recovery rate within condensates
ns
Δ550 EYFP
Δ457 EYFP
Δ550b EYFP
WT EYFP

Figure S7: Molecular characteristics of FOXN1 variants

(A) ChIP-Seq peaks of wild-type and Δ550 FOXN1 expressed in 4D6 TEC. The Venn diagram shows the number of ChIP-Seq peaks that are identified by only wild-type, only Δ550 FOXN1 and both. (B) Weblogo of the MEME-derived FOXN1-binding site motif for TSS-associated peaks (–5 kb before and 100 bp after TSS), $E = 1.7 \times 10^{-362}$ for the WT peaks and $E=2.5 \times 10^{-258}$or the Δ550 FOXN1 peaks. (C) Enrichment of wild-type FOXN1 and Δ550 FOXN1 ChIP-Seq signals over input in a 4kb region flanking the TSS for genes differentially regulated by wild-type FOXN1. All experiments in (A-C) were performed in biological triplicate (although, as detailed in the methods section, one of the Δ550 samples was excluded from the RNA-sequencing analysis as an outlier (D) Anti-Flag immuno-precipitates of FLAG- tagged Δ550 FOXN1 and Western blot analysis of FLAG- tagged Δ550 and myc-tagged wild-type (WT) FOXN1 expressed in 4D6 cells. Protein detection using anti-myc and anti-flag antibodies (E) Graphs showing intrinsic disorder regions and serine content for WT and Δ550 FOXN1. PrDOS (Protein DisOrder prediction System) disorder confidence score is shown on the y axis and amino acid positions on the x-axis. Confidence score above 0.5 predicts a disordered region. Vertical gray lines designate the presence of a serine at a given amino acid position. The purple bar designates the IDRs, the grey bar the forkhead domain and the red bar the transcriptional activation domain. (F) Box plot showing distribution of serine across ordered and disordered protein regions within WT, Δ550, CBP and YBX1 proteins (G) Fluorescence's recovery rate [arbitrary units (a.u.)/sec] of EYFP-labelled Δ550 and GFP-labelled wild-type FOXN1 outside nuclear condensates.(H) Quantification of nuclear condensates formed by EYFP-labelled Δ550, Δ457, Δ550b, wild-type FOXN1 variants. (I)Fluorescence's recovery rate [arbitrary units (a.u.)/sec] of EYFP-labelled Δ550, Δ457, Δ550b, wild-type FOXN1 nuclear condensates following

photobleaching. Mean and SD is indicated, (G-I) unpaired t-test *<0.05, ** <0.01, ***< 0.001 and ****<0.0001

Figure S8

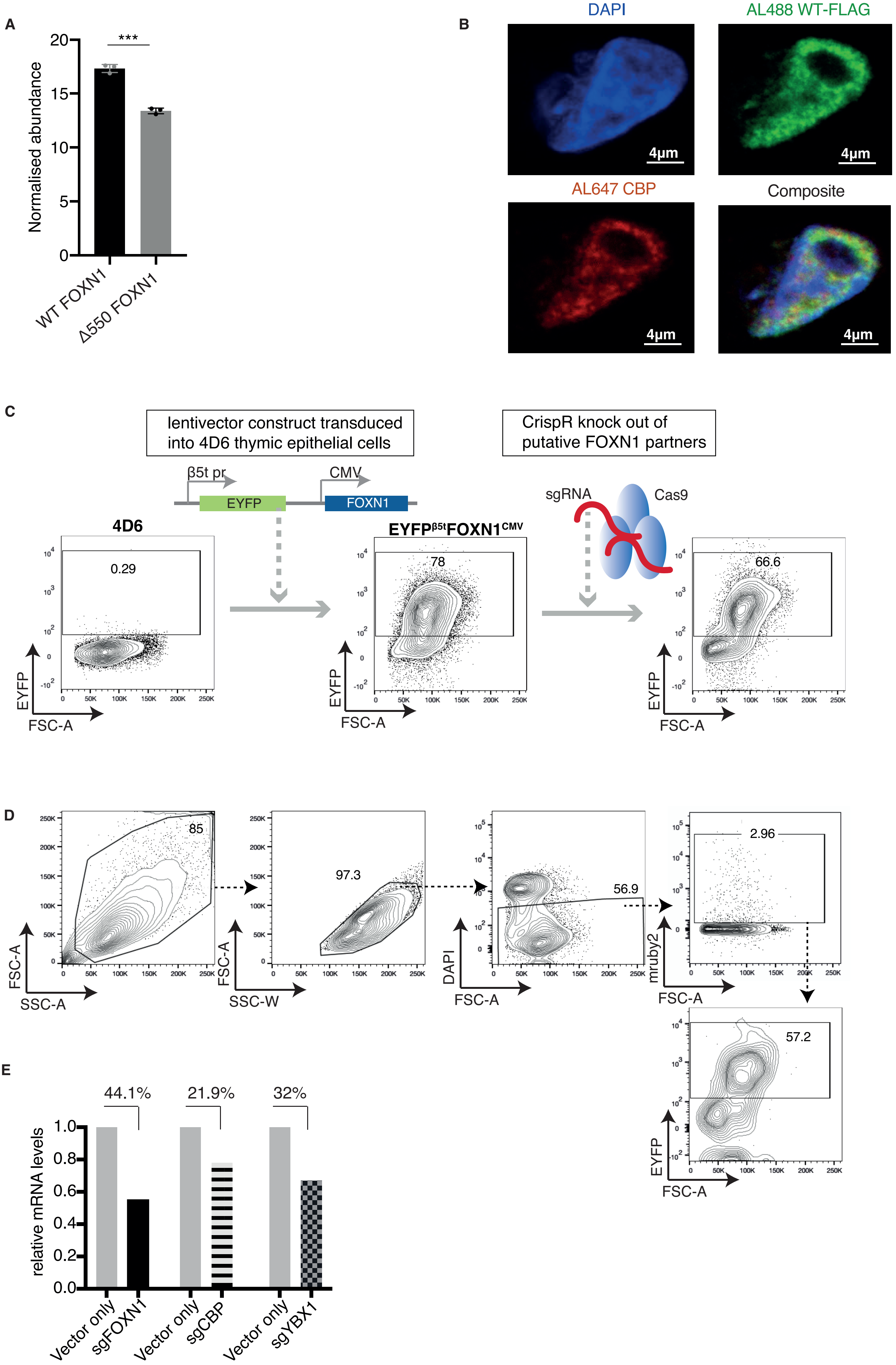

A
Normalised abundance
20
15
10
5
0
***
WT FOXN1
Δ550 FOXN1
B
DAPI
AL488 WT-FLAG
AL647 CBP
Composite
4µm
C
lentivector construct transduced into 4D6 thymic epithelial cells
CrispR knock out of putative FOXN1 partners
β5t pr
EYFP
CMV
FOXN1
sgRNA
Cas9
4D6
EYFPβ5tFOXN1CMV
0.29
78
66.6
EYFP
FSC-A
D
85
97.3
56.9
2.96
57.2
FSC-A
SSC-A
SSC-W
DAPI
mruby2
EYFP
E
44.1%
21.9%
32%
relative mRNA levels
1.0
0.8
0.6
0.4
0.2
0.0
Vector only
sgFOXN1
Vector only
sgCBP
Vector only
sgYBX1

Figure S8: Characterisation of FOXN1 interacting partners

(A) Protein abundance of wild-type or Δ550 FOXN1 in anti-flag pull downs in lysates from 4D6 cells transfected to express wild-type (WT) or Δ550 FOXN1 tagged with a flag. The immunoprecipitates were analysed by Liquid Chromatography with tandem mass spectrometry (LC-MS-MS). Each point corresponds to an individual sample. The data is from a single experiment with three biological replicates per condition. Data shows the mean value and SD, unpaired t-test ***< 0.001 (B) FOXN1 and CBP co-localize in 4D6 cells. Indirect immunofluorescence microscopy combined with DAPI staining and confocal analysis of FLAG-tagged wild-type (WT) FOXN1 and endogenous CBP in 4D6 cells. The extent of CBP and FOXN1 co-localisation as assessed by Pearson's correlation equals to 0.54 (where a value of 0 signifies a lack of colocalization and that of 1 represents complete colocalization) Data is from one independent experiment with 5 biological replicates (C) Schematic representation of the generation of the 4D6-EYFP$^{\beta5t}$ FOXN1^{CMV} reporter cell line used for the validation of candidate proteins interacting with wild-type FOXN1. 4D6 cells were stably transduced to constitutively express FOXN1 under the CMV promoter that in turn controls the transcription of EYFP under the transcriptional control of the *Psmb11* promoter which is a direct target of FOXN1 and drives β5t protein expression. (D) Flow cytometric gating strategy for the identification of EYFP positivity in live 4D6-EYFP$^{\beta5t}$ FOXN1^{CMV} cells that have been transfected with guide RNAs as identified by mruby2 positivity (E) qRT-PCR analysis showing the efficiency of CRISPR mediated deletion in 4D6-EYFP$^{\beta5t}$ FOXN1^{CMV} cells transfected with guide RNAs for *Foxn1*, *Cbp* and *Ybx1*. mRNA levels for *FOXN1, CBP* and *YBX1* were quantified in sorted live, mRuby2 positive cells and compared to *GAPDH* transcripts for normalisation. The mRNA levels for *FOXN1, CBP* and *YBX1* in cells transfected with the Cas9-2A-mRuby2 vector only, were set to an arbitrary value of 1. The numbers above each bar in the graph show the extent of the reduction in the mRNA levels of

each of the genes tested relative to cells transfected with the Cas9-2A-mRuby2 vector only. Data is from one independent experiment with one biological replicate per sample and three technical PCR replicates. The graph shows the mean of the ΔCt from the three technical PCR replicates. .

Figure S9

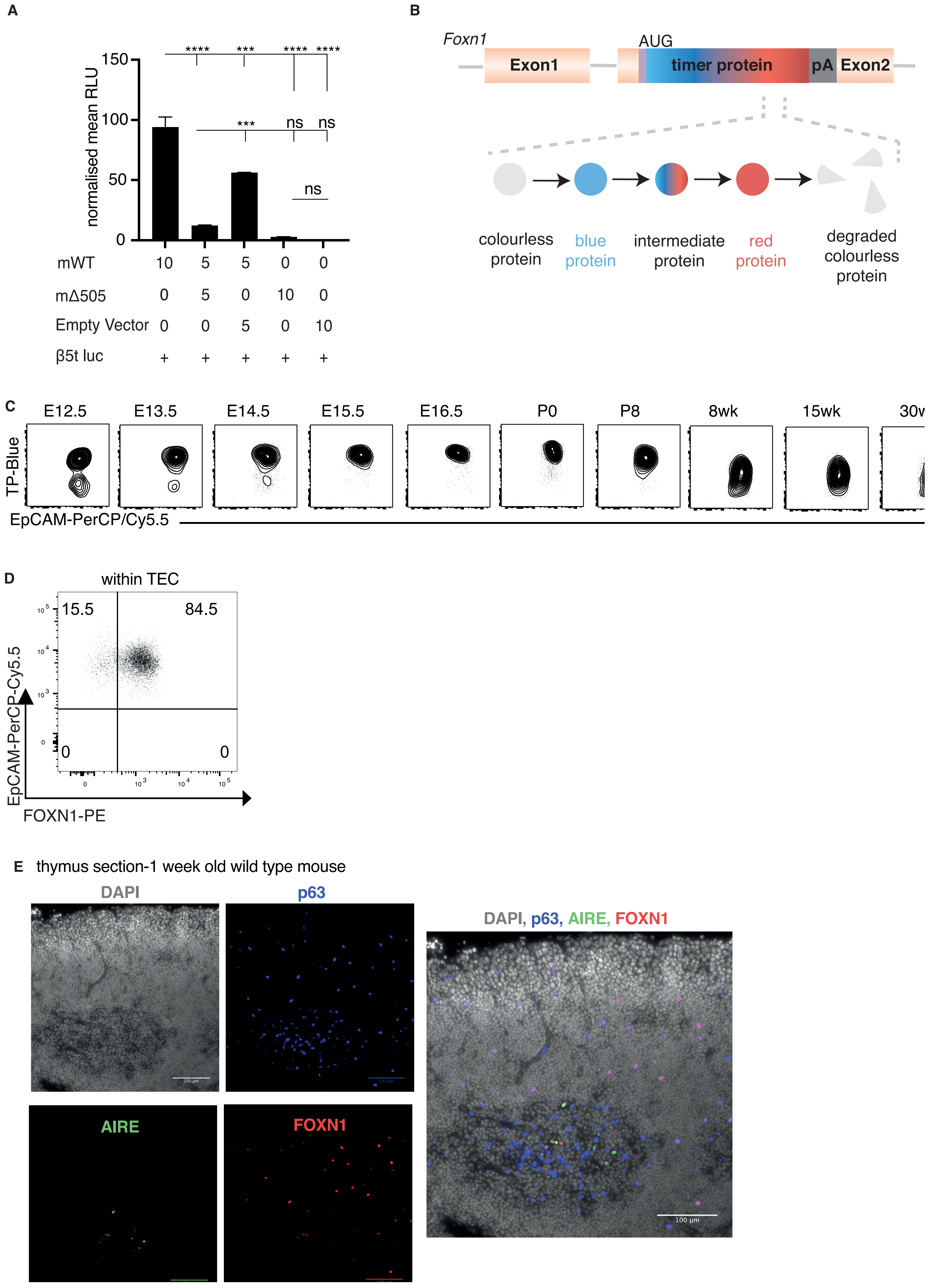
A
normalised mean RLU
150
100
50
0
****
***
****
****
***
ns
ns
ns
mWT 10 5 5 0 0
mΔ505 0 5 0 10 0
Empty Vector 0 0 5 0 10
β5t luc + + + + +
B
Foxn1
Exon1
AUG
timer protein
pA
Exon2
colourless protein
blue protein
intermediate protein
red protein
degraded colourless protein
C
E12.5
E13.5
E14.5
E15.5
E16.5
P0
P8
8wk
15wk
TP-Blue
EpCAM-PerCP/Cy5.5
D
within TEC
15.5
84.5
0
0
EpCAM-PerCP-Cy5.5
FOXN1-PE
E thymus section-1 week old wild type mouse
DAPI
p63
AIRE
FOXN1
DAPI, p63, AIRE, FOXN1
100 µm

Figure S9: FOXN1 function and expression

(A) Expression of a luciferase reporter under the transcriptional control of the *Psmb11* promoter (β5t luc) in TEC1.2 cells transfected with indicated expression vectors. Constitutive renilla expression was used in each transfectant as an internal control and reporter activity was measured as relative light units (RLU) following correction. mWT: mouse wild-type, m Δ505: mouse Δ505 FOXN1. The data is from 3 independent experiments with each 3 technical replicates. Mean value and SD are shown and were and statistically compared by two-tailed unpaired t-test: ≥0.05 (ns), ***< 0.001 and ****<0.0001 (B) Schematic representation of the timer protein construct. (C) representative flow cytometry plots of FTP-blue versus EpCAM in total TEC isolated from the FTP^{FOXN1} mice (D) representative flow cytometry plots of EpCAM versus FOXN1 in total TEC isolated from wild-type mice (E) Immunofluorescent analysis of thymic section from 1 week old wild-type mice for the expression of FOXN1, AIRE and p63. DAPI was used to counterstain cell nuclei.

Table S1 (has been submitted independently as an excel file): Gene ontology analysis of biological processes in the indicated TEC subtypes. Enrichment in $FOXN1^{WT/WT}$ TEC. Data is from 3 $FOXN1^{WT/WT}$ and 3 $FOXN1^{WT/}$Δ505 mice at 5 and 16 weeks of age, with 22,228 interypical TEC analysed.

Table S2 (has been submitted independently as an excel file): Gene ontology analysis of biological processes in the indicated TEC subtypes. Enrichment in in $FOXN1^{WT/\Delta505}$ TEC. Data is from 3 $FOXN1^{WT/WT}$ and 3 $FOXN1^{WT/}$Δ505 mice at 5 and 16 weeks of age, with 22,228 interypical TEC analysed.

Supplementary Table 3

Accession	Gene Name	Subcellular location [CC]	Gene ontology
P17096	HMGA1	Nucleous	DNA Binding [GO:0003677], negative regulation of transcription [GO:0045892]; positive regulation of transcription [GO:0045944];
P12956	XRCC6	Nucleous	DNA Binding [GO:0003677]; positive regulation of transcription [GO:0045944];
Q92793	CBP	Nucleous	acetyltransferase activity [GO:0016407]; RNA polymerase II transcription factor binding [GO:0001085]; transcription coactivator activity [GO:0003713]; transcription corepressor activity [GO:0003714]; transcription factor binding [GO:0008134]; DNA Binding [GO:0003677]
P06748	NPM	Nucleous	activating transcription factor binding [GO:0033613]; RNA binding [GO:0003723]; transcription coactivator activity [GO:0003713]; positive regulation of transcription by RNA polymerase II [GO:0045944];
Q14103	HNRPD	Nucleous, cytoplasm	mRNA splicing GO:0000398,
Q13283	G3BP1	Nucleous, cytoplasm	DNA Binding [GO:0003677]
P78527	PRKDC	Nucleous	DNA Binding [GO:0003677], positive regulation of transcription [GO:0045944];
Q7KZF4	SND1	Nucleous	RNA binding [GO:0003723]; transcription coregulator activity [GO:0003712]
Q8NC51	PAIRB	Nucleous	RNA binding [GO:0003723]
P68104	EF1A1	Nucleous	translation [GO:0006412];
P67809	YBX1	Nucleous, cytoplasm	mRNA splicing GO:0000398, negative regulation of transcription [GO:0000122]; transcription by RNA polymerase II [GO:0006366]
Q6AHZ1	Z518A	Nucleous	DNA Binding [GO:0003677]
Q12906	ILF3	Nucleous	DNA binding [GO:0003677];
P04908	H2A1B	Nucleous	DNA binding [GO:0003677];
O15353	FOXN1	Nucleous	DNA Binding [GO:0003677]
Q96AE4	FUBP1	Nucleous	positive regulation of gene expression [GO:0010628]; transcription by RNA polymerase II [GO:0006366], DNA Binding [GO:0003677]
P09651	ROA1	Nucleous	mRNA splicing GO:0000398
Q05639	EF1A2	Nucleous	translation [GO:0006412];
Q96T23	RSF1	Nucleous	negative regulation of transcription [GO:0045892]; positive regulation of transcription by RNA polymerase II [GO:0045944]; acetyltransferase activity [GO:0016407];
P43243	MATR3	Nucleous	RNA binding [GO:0003723]
P04792	HSPB1	Nucleous	RNA binding [GO:0003723]
O75150	RNF40	Nucleous	mRNA 3'-UTR binding [GO:0003730];
Q9NZI8	IF2B1	Nucleous	RNA binding [GO:0003723]
P11940	PABP1	Nucleous	mRNA splicing GO:0000398
P52272	HNRPM	Nucleous	mRNA splicing GO:0000398
P51991	ROA3	Nucleous	mRNA splicing GO:0000398
Q8N684	CPSF7	Nucleous	mRNA splicing GO:0000398
P48634	PRC2A	Nucleous	RNA binding [GO:0003723]
P31942	HNRH3	Nucleous	mRNA splicing GO:0000398
Q9H869	YYAP1	Nucleous	regulation of cell cycle [GO:0051726]
Q8IV63	VRK3	Nucleous	ATP binding [GO:0005524]; protein phosphatase binding [GO:0019903]; protein serine/threonine kinase activity [GO:0004674]
P42226	STAT6	Nucleous	DNA-binding transcription activator activity [GO:0001228]; [positive regulation of transcription [GO:0045944];

Table S3: List of putative FOXN1 binding partners. Uniprot KB was used to determine the subcellular localisation and the gene ontology (GO) of each protein. VRK3 and STAT6 are the two proteins that inversely correlate with the amount of FOXN1.

Supplementary Table 4

Antibody	Clone	Conjugate	Source
CCR7	4B12	BV421	BD Biosciences
CD11b	M1/70	biotin	BioLegend
CD11c	N418	biotin	BioLegend
CD177 (c-kit)	2B8	APC	Biolegend
CD19	1D3	biotin	eBioscience
CD1d	tetramer	PE	University of Birmingham
CD24	M1/69	APC	BioLegend
CD24	M1/69	PerCP eFluor710	eBioscience
CD25	PC61.5	BV605	BioLegend
CD25	PC61.5	BV421	eBioscience
CD4	RM4-5	PE Texas Red	BioLegend
CD4	RM4-5	APC-Cy7	BioLegend
CD4	RM4-5	FITC	BioLegend
CD44	IM7	PE Cy7	eBioscience
CD44	IM7	PE Texas Red	eBioscience
CD45	30-F11	Alexa Fluor 700	BioLegend
CD49b	DX5	biotin	BioLegend
CD5	53-7.3	PerCP, Cy5.5	BioLegend
CD69	H1.2F3	PE Cy5	BioLegend
CD71	RI7217	PE Cy7	BioLegend
CD80	16-10A1	PE Cy5	BioLegend
CD86	GL-1	PE Cy7	BioLegend
CD8a	53-6.7	Alexa Fluor 700	BioLegend
Cleaved casp 3 (Asp175)	D3E9	AF647	Cell signaling
EPCAM	G8.8	APC-CY7	BioLegend
EPCAM,	G8.8	PerCP Cy5.5	BioLegend
F4/80	BM8	biotin	BioLegend
FOXN1	-	PE	a kind gift by HR Rodewahl[24]
FoxP3,	FJK-16s	eF450	eBioscience
Gp2	2F11-C3	FITC	MBL
Gr1	RB6-8C5	biotin	BioLegend
H2Kb (MHCI)	AF6-88.5	PE	BioLegend
Ly51	6C3	PE	BioLegend
MHC-II	28-14-8	BV421	eBioscience
NK1.1	PK136	biotin	BioLegend
Streptavidin	-	BV605	BioLegend
TCRβ	clone H57-597	APC-Cy7	BioLegend
TCRβ	H57-597	PE	eBioscience
TCRβ	H57-597	FITC	eBioscience
TCRγδ	UC7-13D5	FITC	BioLegend
TCRγδ	GL3	biotin	eBioscience
TER119	TER119	biotin	BioLegend
Tspan8	657909	APC	R&D Systems

Table S4: list of antibodies used for the FACs phenotypic analysis and FACs cell sorting.
